# Posttranslational Modifications of p62/SQSTM1 in Health and Disease

**DOI:** 10.1002/mco2.70949

**Published:** 2026-09-30

**Authors:** Weikai Wang, Xin Jiang, Runing Qiu, Chandra Sugiarto Wijaya, Miaoqin Chen, Jin Wang, Liyuan Zhu, Hongchuan Jin, Lifeng Feng

**Affiliations:** ^1^ Department of Medical Oncology Zhejiang Key Laboratory of Multi‐omics Precision Diagnosis and Treatment of Liver Diseases School of Medicine Cancer Center of Zhejiang University Sir Run Run Shaw Hospital Zhejiang University Hangzhou Zhejiang China; ^2^ Department of Pathology School of Medicine Sir Run Run Shaw Hospital Zhejiang University Hangzhou Zhejiang China

**Keywords:** autophagy, disease, homeostasis, p62/SQSTM1, posttranslational modifications, signal transduction, therapeutic target

## Abstract

p62/SQSTM1 is a multifunctional adaptor protein that serves as a central hub integrating cellular stress responses, including selective autophagy, antioxidant defense, metabolism, and immune signaling. Its diverse functions are precisely orchestrated by a complex network of posttranslational modifications (PTMs) that dynamically regulate its conformation, protein interactions, stability, and subcellular localization. This review provides a comprehensive overview of the p62 PTMs landscape, encompassing phosphorylation, ubiquitination, acetylation, methylation, and emerging cysteine‐based modifications. We first discuss how these PTMs coordinately maintain cellular homeostasis by fine‐tuning autophagic flux, activating antioxidant programs, coordinating nutrient sensing, preserving genomic stability, and regulating immune responses. We then examine how dysregulation of this PTMs network drives pathogenesis in major diseases including cancer, neurodegenerative disorders, metabolic diseases, and infections, with emphasis on context‐dependent molecular mechanisms. Furthermore, we summarize current preclinical evidence targeting p62 PTMs. Finally, we emphasize the critical need for advanced technologies to decipher PTMs crosstalk and dynamic regulation, coupled with the development of corresponding therapeutic strategies, to advance precision medicine. Understanding the p62 PTMs code offers promising opportunities for developing novel biomarkers and targeted therapies for human diseases.

## Graphical abstract

Schematic illustration of p62 as a posttranslational modifications (PTMs)‐programmed integrator in homeostasis, pathogenesis, and therapeutic targeting. (Left) Homeostasis: In physiological states, p62, through various PTMs such as phosphorylation, ubiquitination, and acetylation, coordinates essential cellular functions. These include selective autophagy, antioxidant responses, metabolic regulation, genomic stability, immune activation, and organelle homeostasis. (Middle) p62 as the PTM‐programmed integrator: The diagram illustrates the domain architecture of p62 (PB1, ZZ, TB, LIR, KIR, and UBA) and its extensive PTM landscape, including ubiquitination (Ub), acetylation (Ac), phosphorylation (P), S‐nitrosylation (SNO), methylation (Me), disulfide bonds (S‐S), and S‐acylation (S‐acyl). Notably, multiple upstream kinases (e.g., mTORC1, ULK1, AMPK, TAK1, CK1, and PKC‐δ) phosphorylate specific residues, such as S349 and S403, to dynamically fine‐tune its downstream signaling pathways. Meanwhile, residue K420 is subject to concurrent PTMs (ubiquitination and acetylation), underscoring the intricate crosstalk between distinct modifications at a single site. (Right) Pathogenesis: Aberrant p62 PTM patterns contribute to a wide array of pathological processes. These include cancer (promoting proliferation and chemoresistance), neurodegeneration, metabolic disorders, infectious diseases, inflammatory and autoimmune diseases, as well as muscle disorders, cardiac aging, ischemic diseases, and renal fibrosis. (Bottom) Therapeutic targeting: Translational strategies targeting p62 encompass three main avenues: (1) Biomarker, utilizing specific PTMs (e.g., p‐S349) as disease diagnostic indicators; (2) interventions, employing synergistic, bidirectional, or sequential regulatory modalities for p62 PTM targeted therapy; and (3) precision medicine, tailoring personalized therapeutic regimens based on individual patient p62 PTM profiles.

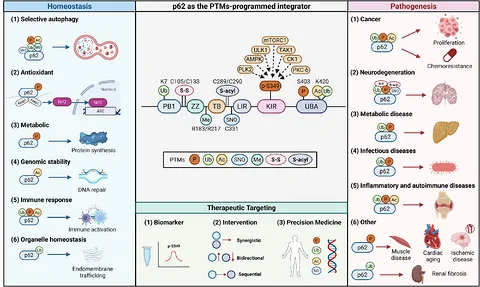

## 1 Introduction

p62/SQSTM1 (hereafter referred to as p62), also known as Sequestosome 1 and homologous to A170 and zeta‐interacting protein (ZIP) in murine species, is a ubiquitously expressed multiple‐function adaptor protein, functioning as a signaling hub and a regulator of homeostasis in cells. And its dysregulation has been implicated in pathogenesis including cancer, neurodegenerative disorders, and metabolic diseases. Its research trajectory exemplifies a profound evolution in the understanding of its biological functions. Initially identified over two decades ago as a phosphotyrosine‐independent ligand for the Src homology 2 (SH2) domain of the lymphocyte‐specific protein tyrosine kinase (Lck) [1], the biological significance of p62 has since undergone several fundamental revisions. Key milestone discoveries have driven this conceptual advancement. The identification of its ubiquitin‐associated (UBA) domain first linked p62 to the ubiquitination system. The elucidation of the microtubule‐associated protein 1A/1B‐light chain 3 (LC3)‐interacting region (LIR) established its central role as a key adaptor protein for selective autophagy. Furthermore, the characterization of the Kelch‐like ECH‐associated protein 1 (Keap1)‐interacting region (KIR) revealed a novel mechanism through which p62 activates the nuclear factor erythroid 2‐related factor 2 (Nrf2) antioxidant pathway via autophagic degradation of Keap1. Beyond the direct functions of these discrete domains, more critical conceptual breakthroughs include the discovery that p62 undergoes liquid–liquid phase separation [2], enabling it to dynamically assemble membraneless organelles and regulate signal transduction. Additionally, its function as a scaffold for mechanistic target of rapamycin complex 1 (mTORC1) activation on the lysosome solidified the pivotal role of p62 in cellular nutrient sensing and metabolic regulation [3, 4]. Collectively, these discoveries have shaped the modern paradigm of p62 as a multifunctional hub integrating diverse signaling pathways, including selective autophagy [5, 6, 7, 8, 9], antioxidant defense [10, 11], and metabolism [3, 4], thereby orchestrating adaptive cellular responses to environmental stress.

p62 is a 440‐amino acid, multidomain scaffold protein whose modular architecture constitutes the molecular basis for its diverse biological functions. The N‐terminal Phox and Bem1 (PB1) domain mediates either self‐oligomerization of p62 or its interaction with other PB1 domain‐containing proteins, such as protein kinase C zeta (PKCζ) and mitogen‐activated protein kinase kinase 5 (MEK5), which is central to p62's ability to form higher‐order complexes and function as a scaffold [12]. The centrally located ZZ‐type zinc finger (ZZ) domain and the TNF receptor‐associated factor 6 (TRAF6)‐binding domain collectively mediate its activation of the nuclear factor kappa‐light‐chain‐enhancer of activated B cells (NF‐κB) signaling pathway; the former interacts with receptor‐interacting protein kinase 1 (RIP1), while the latter directly recruits TRAF6 [13]. The adjacent KIR is a key regulatory module. It binds to Keap1, a component of the Cullin 3 E3 ligase complex, and promotes the autophagic degradation of Keap1. This process relieves Keap1‐mediated inhibition of the transcription factor Nrf2, thereby enabling the noncanonical activation of the Nrf2 signaling pathway under stress conditions [10, 11]. The C‐terminal region of p62 is critical for its role in selective autophagy. The UBA domain specifically recognizes and binds ubiquitinated substrates [14]. Structural studies on UBA domains of related autophagy receptors reveal that their dynamic, reversible, and regulated interaction with ubiquitin chains underlies their function as ubiquitin sensors [15]. The LIR domain binds to members of the LC3/GABA type A receptor‐associated protein (GABARAP) protein family on the autophagosomal membrane, thereby tethering the cargo for degradation [16, 17]. Furthermore, p62 contains two classical monopartite nuclear localization signals (NLS) and a leucine‐rich nuclear export signal (NES), suggesting active nucleocytoplasmic shuttling. This trafficking may be relevant to its potential functions in processes such as transcriptional regulation [18]. The domain organization and functions of p62 are summarized in Figure 1. For a more detailed discussion of its domains and their regulated signaling pathways, readers are referred to authoritative reviews by Moscat et al., and Chen et al., [19, 20].

**FIGURE 1:**
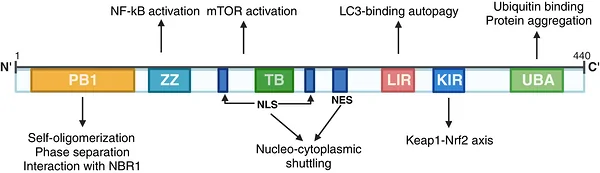
Schematic representation of the domain structure and multifunctional nature of human p62. The linear representation of the p62 protein highlights its major functional domains with approximate amino acid boundaries indicated above. The N‐terminal PB1 domain mediates self‐oligomerization and phase separation and facilitates interaction with NBR1. The ZZ‐type zinc finger domain is involved in NF‐κB activation. The noncanonical region between the ZZ and TB domains is responsible for the mTOR pathway activation. The nuclear localization signal (NLS) and nuclear export signal (NES) within the protein regulate its nucleo‐cytoplasmic shuttling. The central region contains the LIR (LC3‐interacting region) motif, which is responsible for LC3 binding and autophagy, and the KIR (Keap1‐interacting region) motif, which governs the Keap1–Nrf2 axis. The C‐terminal UBA domain (ubiquitin‐associated domain) binds ubiquitin and mediates protein aggregation.

Therefore, the modular architecture of p62 provides the essential static structural foundation for its functions. However, to fully understand how p62 acts as a highly dynamic signaling integrator capable of precisely responding to complex and variable cellular cues, it is crucial to decipher the regulatory language governing its functional activity—namely, posttranslational modifications (PTMs). PTMs do not merely serve as static labels but constitute a sophisticated and dynamic regulatory system [21, 22, 23]. Recent studies have profoundly revealed that diverse PTMs, such as phosphorylation, ubiquitination, and acetylation, function as versatile molecular switches [24, 25, 26]. Through reversible modifications of specific amino acid residues, they dynamically reprogram the functional status of p62, including its protein‒interaction network, conformation, stability, and subcellular localization. This dynamic layer of PTMs regulation enables p62 to transcend its static blueprint and adapt flexibly to varying physiological or stress conditions. Critically, the precision of this PTM network directly dictates the dual role of p62 in maintaining cellular health and driving pathological processes. Dysregulation of this network has emerged as a hallmark of various human diseases, including cancer and neurodegenerative disorders.

Research into the PTMs of p62 began in the 1990s. As early as 1997, studies identified p62 as a phosphoprotein in mouse macrophages and demonstrated that kinases such as casein kinase 2 (CK2) and protein kinase A (PKA) could phosphorylate it in vitro, although its biological functions remained unclear at the time [27]. Subsequent work has progressively expanded this initial focus on phosphorylation (e.g., TANK‐binding kinase 1 (TBK1)‐ and mTORC1‐mediated phosphorylation in autophagy [11, 28, 29]) toward a far more comprehensive PTMs landscape. This landscape now encompasses diverse validated p62 PTMs, including ubiquitination [30], acetylation [2], methylation [31], ubiquitin‐like modifications (e.g., FAT10ylation [32]), and various cysteine modifications (such as S‐nitrosylation [33, 34] and S‐acylation [35]). By fine‐tuning p62's interactome, conformation, stability, and localization, these PTMs enable its participation in diverse critical processes, including selective autophagy, antioxidant stress response, cell cycle regulation, metabolic adaptation, DNA damage repair, and immune signaling. Notably, extensive crosstalk exists among different PTM types, forming a dynamic regulatory network that plays a central role in various pathological states, including cancer, neurodegenerative disorders, and metabolic diseases. This continually expanding PTMs map not only deepens our understanding of p62's functional versatility but also opens new avenues for targeted therapies in related diseases. However, fundamental challenges remain in this field. First, previous research perspectives are often fragmented, predominantly focusing on the role of p62 PTMs (even a single PTM) within an isolated pathway (such as a recent review focusing on p62 PTMs and their counter‐regulation in the Keap1–Nrf2/autophagy axis [36]) while overlooking the broader crosstalk between different modifications. This approach has limited our ability to decipher the synergistic or antagonistic regulatory logic at a systems level. Second, our current understanding of how these PTMs evolve dynamically within specific pathological contexts (such as tumor metabolic reprogramming or neuroinflammation) and their precise pathophysiological significance remains quite limited. Furthermore, the specific biological functions and regulatory mechanisms of several newly identified PTMs, such as methylation and cysteine modifications, await thorough exploration.

In light of this, the present review aims to systematically delineate and prospect the landscape of p62 PTMs from an integrative perspective of health and disease. Moving beyond a mere catalog of modification events, we endeavor to construct a dynamic, physiology‐, and pathology‐driven regulatory network of p62 PTMs. The review will first elucidate how PTMs fine tune p62 to maintain cellular homeostasis. It will then focus on dissecting how dysregulation of the PTMs network drives pathogenesis in specific disease contexts, such as cancer and neurodegenerative disorders, bridging preclinical models with clinical evidence. Finally, we will outline future research directions to provide a forward‐looking theoretical foundation for developing therapeutic strategies targeting p62 PTMs.

## 2 The Role of the p62 PTMs Regulatory Network in Maintaining Cellular Homeostasis

### 2.1 Precise Regulation of the Entire Autophagy Cascade by p62 PTMs

Autophagy is a multistep catabolic process, beginning with induction and nucleation, followed by substrate recognition, autophagosome elongation and closure, and culminating in the degradation of cargo via autophagosome–lysosome fusion [37, 38, 39]. A central challenge in selective autophagy is the specific recognition of ubiquitinated substrates (such as misfolded proteins or damaged organelles) and their precise targeting to autophagosomal engulfment. The core autophagy adaptor protein p62 serves as the molecular hub addressing this challenge. It captures substrates via its UBA domain and tethers them to LC3 proteins on the autophagosomal membrane through its LIR [40, 41]. However, the function of p62 is far from a static tether. Its substrate recognition and binding, oligomerization and phase separation, docking to the autophagosomal membrane, and its own protein turnover are all dynamically regulated by a sophisticated PTMs network (Figure 2). This network enables p62 to integrate diverse cellular signals, thereby allowing flexible switching between basal maintenance and stress‐adaptive states, which ensures precise control over autophagic flux.

**FIGURE 2:**
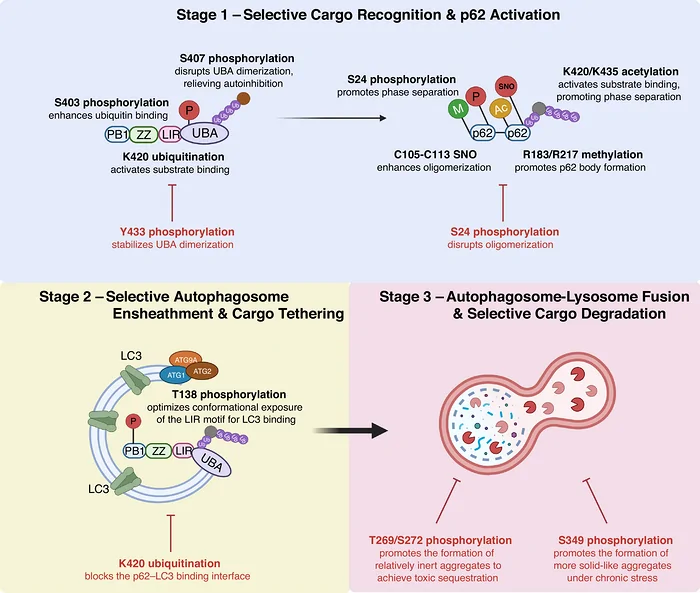
p62 PTMs network orchestrates selective autophagy. S403 phosphorylation enhances ubiquitin binding; S407 phosphorylation relieves UBA autoinhibition; K420 ubiquitination activates substrate binding, while S24 phosphorylation, K420/K435 acetylation, R183/R217 methylation, and C105–C113 S‐nitrosylation promote oligomerization and liquid–liquid phase separation to form p62 bodies. T138 phosphorylation exposes the LIR to optimize LC3 engagement while ATG9A–ATG2 support membrane growth. Cargo‐filled autophagosomes fuse with lysosomes for degradation. Flux‐braking checkpoints include S24 phosphorylation and Y433 phosphorylation limiting oligomerization or stabilizing UBA dimerization, K420 ubiquitination blocking the p62–LC3 interface, T269/S272 phosphorylation promoting inert aggregation for toxic sequestration, and S349 phosphorylation favoring inclusion‐body‐like aggregates under chronic stress.

To precisely initiate the selective autophagy program, cells converge various upstream signals onto the hub protein p62. Through its complex PTMs network, p62 efficiently translates initiation signals into substrate clearance.

Under energy stress or proteotoxic stress, the cellular autophagy program is activated by upstream kinases such as AMP‑activated protein kinase (AMPK), which in turn activates UNC‑51‑like kinase 1 (ULK1). The ordered phosphorylation of the core adaptor p62 by ULK1 then serves as a key mechanism to translate this activation into substrate clearance. ULK1 first phosphorylates p62 at Serine 407 (S407), which disrupts the dimerization of its UBA domain. This releases auto‐inhibition and clears steric hindrance, permitting efficient subsequent phosphorylation at Serine 403 (S403) [42]. The phosphorylation of S403 then directly enhances p62's ability to recognize and recruit ubiquitinated substrates [43]. This S407 “de‐repression” and S403 “activation” form a positive feedback loop that amplifies the signal. Critically, the resultant dual phosphorylation induces a specific conformation in the UBA domain, enabling high‐affinity, precise recognition of ubiquitin chain epitopes. Thus, through this ULK1‐mediated cascade, p62 is converted into a high‐fidelity ubiquitin sensor, capable of executing precise substrate clearance [44].

Other kinases, such as CK2 and AMPK, can also phosphorylate S403 under different stresses (e.g., proteotoxicity, hypoxia), ensuring efficient execution of p62‐mediated substrate recognition and recruitment across various pressure conditions [45, 46]. During nutrient deprivation, acetylation of Lysine 420 (K420) and Lysine 435 (K435), mediated by Tat‐interactive protein 60 kDa (TIP60), not only enhances ubiquitin binding by disrupting UBA dimerization, but more critically, drives liquid–liquid phase separation to form dynamic p62 bodies, thereby efficiently concentrating dispersed substrates [2]. Similarly, protein arginine methyltransferase 6 (PRMT6)‐mediated methylation of Arginine 183/217 (R183/R217) [31] and transforming growth factor‐β‐activated kinase 1 (TAK1)‐mediated phosphorylation of Serine 24 (S24) [47] also positively promote this p62 body formation via phase separation. Notably, modification at K420 is not limited to acetylation. Under conditions such as ubiquitin stress, the E3 ubiquitin ligase Keap1/Cullin 3 complex or the E2 enzymes ubiquitin‐conjugating enzyme E2 D2/3 (UBE2D2/3) can also catalyze ubiquitination at this site, similarly activating p62's substrate‐binding capacity by disrupting UBA dimerization [48, 49]. Furthermore, under oxidative stress, the formation of an intermolecular disulfide bond between Cysteine 105 and Cysteine 113 (C105–C113) in p62 directly enhances its oligomerization and promotes the recruitment of ubiquitinated substrates, thereby accelerating the initiation of autophagic flux [50]. These physically distinct condensates, driven by diverse modifications, provide a platform for the subsequent precise assembly of autophagosomes. Following substrate enrichment, polyubiquitin chain‐p62 condensates can directly drive the recruitment of core autophagy machinery, such as the autophagy‐related protein 9A (ATG9A)–autophagy‐related protein 2 (ATG2) complex, to initiate autophagosome membrane formation [51]. During this process, the effective docking of p62 to the autophagosomal membrane protein LC3 is also precisely gated by its own conformational regulation to ensure efficient substrate loading. In its basal state, the N‐terminal PB1 and ZZ domains of p62 jointly constrain the exposure of the LC3‐binding LIR motif. However, phosphorylation of Threonine 138 (T138) within the ZZ domain releases this conformational constraint, thereby optimizing the interaction between p62 and LC3 on the nascent autophagosomal membrane [52].

To prevent excessive autophagy and resource depletion, cells employ a sophisticated PTMs network to negatively regulate p62 function through multiple dimensions. First, the fundamental ability of p62 to oligomerize is itself finely tuned by PTMs, with effects highly dependent on the upstream signaling context. For example, under basal conditions with active growth signals, PKA‑mediated phosphorylation of S24 disrupts PB1 domain‑mediated interactions, thereby inhibiting p62 oligomerization and its autophagic function [53, 54]. Beyond regulating oligomerization, cells also directly suppress p62's substrate‑binding or delivery capacity through modifications. Under active growth signaling, epidermal growth factor receptor (EGFR)‑mediated phosphorylation of Tyrosine 433 (Y433) stabilizes the dimerization of the p62 UBA domain, which instead inhibits its binding to ubiquitinated substrates, blocking autophagic cargo delivery at the source [55]. A more specific termination mechanism is provided by the E3 ligase tripartite motif‐containing protein 38 (TRIM38), which catalyzes a unique K63‑linked polyubiquitination at the K420 site of p62. This modification does not target p62 for degradation; rather, it creates steric hindrance that physically shields the p62–LC3 interaction interface, thereby completely severing the autophagic flux [56]. Under extreme stress, cells can even reprogram the core function of p62. During sustained proteasome inhibition, phosphorylation of Threonine 269 and Serine 272 (T269/S272) by the p38δ kinase inhibits the prodegradative S403 phosphorylation and drives p62 to encapsulate toxic proteins into relatively inert aggregates. This represents a functional switch from promoting “degradation” to mediating “sequestration” as an emergency protective strategy [57, 58]. Similarly, Polo‐like kinase 2 (PLK2)‑mediated phosphorylation of Serine 349 (S349) under prolonged stress also tends to favor the formation of such aggregates rather than efficient autophagic degradation [59]. Even more complex, when the autophagic degradation pathway itself is impaired (e.g., due to defects in autophagosomal membrane closure), accumulated unclosed precursor membranes aberrantly enrich and activate TBK1, leading to robust phosphorylation of p62 at S403. However, in the context of a blocked degradation pathway, this phosphorylation does not initiate classical degradation but instead drives p62 to form large, insoluble aggregates with ubiquitinated cargo, achieving physical containment of toxic material [60]. This series of functional switches from “degradation” to “sequestration” serves as an emergency protective strategy and vividly exemplifies the extreme functional plasticity of the p62 PTMs network, whose downstream outcome is strictly dictated by the upstream signaling context and the functional state of organelles. Furthermore, feedback regulation from upstream autophagy machinery also participates in this network. The autophagy‑initiating protein FAK family kinase‐interacting protein of 200 kDa (FIP200) can restrict the excessive recruitment of TBK1 to p62 condensates, thereby setting an activation threshold for S403 phosphorylation and preventing aberrant or excessive autophagy activation [61].

Furthermore, the PTMs network of p62 contributes to maintaining autophagic homeostasis by regulating its own protein abundance. The protein level of p62 is dynamically controlled through ubiquitination events centered on the Lysine 7 (K7) site: the E3 ligase tripartite motif‐containing protein 25 (TRIM25) catalyzes ubiquitination at K7 and Lysine 189 (K189), promoting p62 degradation, whereas the deubiquitinase ubiquitin‐specific protease 13 (USP13) removes ubiquitin chains from K7, thereby stabilizing p62. These two opposing mechanisms function antagonistically to maintain a homeostatic pool of p62 within the cell, providing critical protein‐level support for basal autophagy activity [62, 63].

### 2.2 Priming and Activation of Physiological Antioxidant Defense

Cells continuously generate reactive oxygen species (ROS) during normal metabolism and under physiological stress. This endogenous oxidative pressure poses a potential threat to biomolecules while simultaneously serving as an important source of signaling cues [64]. Consequently, cells have evolved a sophisticated system to sense, respond to, and neutralize ROS, thereby maintaining the core equilibrium of redox homeostasis. Within this system, Nrf2‐mediated antioxidant signaling acts as a central defensive hub. In addition to the canonical oxidative stress‐induced Keap1 modifications, the noncanonical p62–Keap1–Nrf2 axis also facilitates Nrf2 activation via competitive inhibition of Keap1. Under oxidative and other stress conditions, p62 functions as a key adaptor protein that can competitively bind to Keap1. This binding releases Keap1's inhibition of the transcription factor Nrf2, thereby activating the expression of a battery of antioxidant and detoxifying genes, which systemically enhances cellular defense capacity [10]. The activity of p62–Keap1–Nrf2 axis is precisely governed by a highly dynamic p62 PTMs network, enabling it to dynamically respond to varying characteristics of oxidative stress and thereby precisely convert upstream signals into Nrf2‐mediated transcriptional output. This mechanism achieves accurate control of the antioxidant defense, ultimately maintaining a dynamic equilibrium between the defensive response and oxidative pressure.

Phosphorylation of S349 on p62 serves as a core molecular switch for activating the noncanonical Keap1–Nrf2 antioxidant pathway. When phosphorylated at S349, p62 undergoes a conformational change that transforms it into a high‐affinity decoy for Keap1 [11]. Concurrently, this modification drives p62 to undergo liquid–liquid phase separation, forming dynamic condensates with increased fluidity and enhanced binding affinity for Keap1 [65]. By stably binding to Keap1, phosphorylated p62 competitively occupies the sites on Keap1 originally responsible for recognizing and targeting the transcription factor Nrf2 for poly‐ubiquitination and subsequently proteasome‐dependent degradation. This disrupts the Nrf2‐targeting function of the Keap1–Cullin 3 complex, ultimately leading to Nrf2 stabilization, nuclear translocation, and the initiation of antioxidant gene transcription programs [11]. The activity of this key switch is regulated by a sophisticated, multilayered network with spatiotemporal specificity. At the basal defense level, continuous exogenous stress, such as that encountered in the intestinal epithelium, relies on TAK1‐mediated basal‐level S349 phosphorylation to maintain routine protection [66]. When confronted with acute energy or metabolic challenges like hypoglycemia or lipotoxicity, AMPK can directly phosphorylate p62 S349 [67]. Oxidative stress itself can activate the DNA damage response pathway, where the activated checkpoint kinase 2 (CHK2) kinase phosphorylates both p62 S349 and Nrf2, achieving synergy between genome protection and antioxidant defense [68]. Regarding physiological adaptation, exercise enhances antioxidant protein expression by activating mTORC1‐mediated p62 S349 phosphorylation, constituting a core mechanism for exercise‐induced antioxidant adaptation [69]. Additionally, exercise can drive mouse p62 S351 phosphorylation (corresponding to human S349) by inducing IL‐1β and upregulating the adaptor protein neighbor of BRCA1 gene 1 (NBR1), revealing a more upstream integrated pathway [70]. The sophistication of this regulatory network is further exemplified by its remarkable spatiotemporal specificity. Spatially, kinases such as ULK1 can be directly recruited to the sites of bodies or aggregates formed by p62 and its substrates, enabling on‐site phosphorylation of S349. This precisely confines the antioxidant response to the micro‐domain where damage occurs, achieving targeted repair [24]. Temporally, its dynamic regulation is also extremely rapid. For example, within seconds after acute exhaustive exercise, the phosphorylation level of skeletal muscle p62 S349 significantly increases, and it rapidly diminishes within 1 min after oxygen supply is restored, demonstrating a precise spatiotemporal regulatory capability on the scale of minutes [71].

To prevent the antioxidant response from being overactivated, this pathway is also equipped with a dynamic balance and negative feedback mechanism centered on the p62 K7. Under oxidative stress, the E3 ubiquitin ligase tripartite motif‐containing protein 21 (TRIM21) catalyzes K63‐linked ubiquitination at p62 K7. This modification inhibits the oligomerization of p62 and its binding to Keap1, thereby negatively regulating the Nrf2 pathway and acting as a molecular brake [30]. Interestingly, under lipotoxic stress induced by free fatty acids, TRIM21 itself becomes inactivated due to disulfide bond‐mediated oxidation and oligomerization of its N‐terminal cysteine clusters. This inactivation releases its inhibitory ubiquitination on p62 K7, thereby promoting p62 oligomerization and PB1 domain dimerization via the K7‐D69 hydrogen bond. This dimerization facilitates the recruitment of TBK1, which phosphorylates p62 at S403. S403 phosphorylation in turn promotes p62 aggregation and sequestration of Keap1, thereby activating the Keap1–Nrf2 antioxidant pathway [72]. This reveals an adaptive strategy where cells actively release the brake via a redox mechanism to enhance defense under metabolic stress. Conversely, the deubiquitinase USP13 can remove the ubiquitin chains at the K7 site, enhancing the stability of p62, promoting its oligomerization and autophagic function, and thus positively regulating this pathway [62]. The antagonistic actions of USP13 and TRIM21 constitute a fine‐tuning mechanism for p62 protein levels and pathway activity.

The antioxidant function of p62 is further fine‐tuned by other PTMs sites, which are often triggered under sustained or intense oxidative stress. For instance, in the highly oxidative tumor microenvironment, the ketohexokinase‐A (KHK‐A) kinase phosphorylates p62 at Serine 28 (S28), thereby activating Nrf2 by promoting the p62–Keap1 interaction [73]. In contrast, under oxidative stress in neurons, T138 phosphorylation mediated by the leucine‐rich repeat kinase 2 (LRRK2) kinase impairs p62's ability to activate the Nrf2 pathway [74]. Furthermore, p62 itself can act as a redox sensor: its C105 and C113 residues form intermolecular disulfide bonds when ROS levels increase. This oxidative modification, synergizing with the PB1 domain, significantly promotes p62 oligomerization and recruitment of ubiquitinated substrates, thereby more efficiently activating autophagic flux to promote cell survival [50]. In addition, under nutrient stress conditions such as serum starvation, upregulated RAD23 homolog B (RAD23B) activates the deglycosylating enzyme N‐glycanase 1 (NGLY1), which accelerates the turnover of p62 S349 phosphorylation, thereby fine‐tuning the activation intensity of the Keap1–Nrf2 antioxidant pathway [75].

### 2.3 Coordination of Nutrient Sensing and Anabolism

The PTMs network of p62 plays a central role as a hub in coordinating cellular nutrient sensing and metabolic state transitions. It can mediate anabolic instructions under nutrient sufficiency and initiate protective adaptive programs during nutrient deprivation.

Under nutrient‐sufficient conditions, p62 participates directly in converting signals such as amino acids into anabolic instructions by receiving specific phosphorylation modifications. Among these, the phosphorylation of p62 at T269/S272 is a key step. This modification can be triggered by the mitogen‐activated protein kinase kinase kinase 3 (MEKK3)–mitogen‐activated protein kinase kinase 3/6 (MEK3/6)–p38δ signaling cascade in response to nutrient signals. The phosphorylated p62 acts as a scaffold platform, recruiting TRAF6 to the vicinity of the lysosomal membrane, which in turn activates the mTORC1 complex localized there. This drives anabolic processes such as ribosome biogenesis and protein translation, supporting physiological cell growth [76]. This regulatory mechanism is context dependent. For example, in melanoma, the dual‐specificity tyrosine phosphorylation‐regulated kinase 3 (DYRK3) kinase can phosphorylate p62 T269 more efficiently and drive mTORC1 activation, promoting tumorous growth [77]. It is noteworthy that p62 may achieve a higher level of signal integration in this pathway: the activated mTORC1 can further phosphorylate p62 at S349, and this modification can in turn activate the Keap1–Nrf2 antioxidant pathway [11, 76]. This suggests that through sequential distinct modifications, p62 may prospectively coordinate antioxidant defense while initiating anabolism to cope with the oxidative stress accompanying synthesis, highlighting its foresight and complexity as a signal integration platform.

In contrast to the proanabolic instructions under nutrient sufficiency, under stress conditions of nutrient deprivation (e.g., serum starvation), the PTMs and protein stability network of p62 is reprogrammed to initiate protective adaptive programs. Research on synovial fibroblasts from osteoarthritis patients revealed that phosphorylation of p62 at S349, which is the key signal for activating the Keap1/Nrf2 antioxidant pathway, is rapidly degraded via the proteasomal pathway under resting conditions to maintain homeostasis. However, under serum starvation, the turnover of this modification is significantly accelerated because cells simultaneously activate dual degradation pathways involving both the proteasome and the autophagy–lysosome system [75]. The significance of actively accelerating phosphorylated‐S349‐p62 (p‐S349 p62) turnover during starvation is that by rapidly clearing the p‐S349 p62 that has already exerted its effect, cells can both avoid overactivation of the antioxidant pathway and the resulting depletion of energy and reducing equivalents and maintain the sensitivity of the signaling pathway, ensuring a dynamic response to sustained stress. Concurrently, serum starvation rapidly induces the expression of RAD23B, an adaptor molecule linking the proteasome to the deglycosylase NGLY1. Furthermore, starvation reversibly alters the apparent molecular weight of p62, suggesting that deglycosylation events may be involved in the metabolic reprogramming of p62, although further studies are needed to elucidate its role [75].

In summary, through its dynamically tunable PTMs network, p62 sensitively perceives changes in nutrient status and correspondingly switches its functional roles: acting as a promoter of anabolism during nutrient sufficiency and as a coordinator of survival adaptation during nutrient scarcity. This dual functionality further consolidates the position of p62 as a central integrator of cellular metabolism and stress responses.

### 2.4 Maintenance of Genomic Stability

Beyond its roles in autophagy regulation, antioxidant defense, and metabolic coordination, the PTMs network of p62 extends its functions to maintain genomic integrity. By undergoing precisely regulated modifications, p62 directly participates in DNA damage response pathways, thereby consolidating its role as a key regulator of cellular homeostasis at the genomic level.

In maintaining genomic stability, p62 participates directly in DNA damage repair through specific PTMs. Studies have found that the acetyltransferase human males absent on the first (hMOF) and the deacetylase sirtuin 7 (SIRT7) dynamically regulate the acetylation level of p62 at Lysine 264 (K264). This site is located within its NLS region, and acetylation modification promotes the translocation of p62 into the nucleus. Once inside the nucleus, acetylated p62 interacts with apurinic/apyrimidinic endonuclease 1 (APE1), a key protein in the base excision repair pathway. This interaction not only enhances the binding of APE1 to damaged chromatin but also boosts its endonuclease activity, thereby effectively countering oxidative or alkylating damage to maintain genomic stability and ensure cell survival [26]. However, when faced with extreme, irreversible genotoxic damage, the function of p62 may undergo a fundamental shift. Latest research further reveals that under genotoxic stress such as radiation, p62 can be recruited to micronuclei or abnormal chromatin sites and undergo oligomerization via ROS‐mediated cysteine oxidation (specific site unidentified). This oligomerized p62 can degrade key components of nuclear envelope repair (such as the endosomal sorting complexes required for transport III (ESCRT III) complex) via autophagy and interfere with the choice of DNA repair pathways, thereby exacerbating genomic instability and even leading to catastrophic chromosomal rearrangements, which may subsequently enhance tumor resistance to radiotherapy and drive malignant progression [78].

### 2.5 Regulation of Innate Immunity and Inflammatory Responses

The PTMs network of p62 performs an exquisite bidirectional regulatory function within the immune system. It is integral to both the initiation of innate immune defense and the timely resolution of inflammatory signals, positioning it as a key molecule in maintaining immune homeostasis.

During the immune activation phase, p62 facilitates antimicrobial responses. The kinase TBK1, activated by the E3 ligase tripartite motif‐containing protein 23 (TRIM23), phosphorylates p62 at S403. This forms a TRIM23–TBK1–p62 axis that promotes the selective autophagic clearance of intracellular pathogens [79]. Conversely, to prevent excessive or prolonged immune activation, p62 also mediates negative feedback mechanisms. Following the activation of cytosolic DNA sensing pathways like cyclic GMP‐AMP synthase (cGAS)–stimulator of interferon genes (STING), phosphorylation of p62 facilitates the autophagic degradation of the central adaptor protein STING, thereby attenuating the signaling cascade. Specifically, TBK1‐mediated phosphorylation at S403 [80] and casein kinase 1 (CK1)‐mediated phosphorylation at S349 [81] have both been implicated in this regulatory mechanism. Furthermore, p62 PTMs are involved in directly limiting interferon and inflammatory responses. Research reveals that the E3 ligase tripartite motif‐containing protein 2 alpha (TRIM2α) catalyzes the ubiquitination of p62 at Lysine 91 (K91). This modification promotes p62 oligomerization, which in turn targets the transcription factor interferon regulatory factor 3 (IRF3) for autophagic degradation, thereby preventing excessive and sustained Type I interferon signaling [82]. Similarly, in response to inflammation, the deacetylase sirtuin 1 (Sirt1) can activate mitophagy by regulating the acetylation status of p62. This promotes the clearance of damaged mitochondria and reduces intracellular ROS levels, which is crucial for limiting excessive inflammatory signal transduction [83]. In summary, through its intricate PTMs network, p62 achieves a dynamic balance between the activation and resolution of innate immune and inflammatory responses, underscoring its critical role as an immunoregulatory hub.

### 2.6 Endomembrane Trafficking and Organelle Homeostatic Regulation

The PTMs network of p62 also extends to the fine regulation of the cell's internal logistics system, specifically endomembrane trafficking and the spatial organization of organelles. This function underscores the pivotal role of p62 as a dynamic scaffold in coordinating the homeostasis of intracellular vesicular transport. Its core mechanism involves a sophisticated tether‐release cycle. The E3 ubiquitin ligase RING finger protein 26 (RNF26), located on the endoplasmic reticulum (ER) membrane and cooperating with its specific E2 enzyme ubiquitin‐conjugating enzyme E2 J1 (UBE2J1), can specifically catalyze the ubiquitination of p62 at K435 [84, 85]. Modified in this way, p62 transforms into a ubiquitin‐enriched platform, which subsequently attracts various endosomal vesicle adaptor proteins containing ubiquitin‐binding domains (such as epidermal growth factor receptor pathway substrate 15 (EPS15) and Toll‐interacting protein (TOLLIP)) via its own ubiquitin‐binding domain. Consequently, the RNF26–p62 complex forms a molecular tethering hub around the ER, temporarily sequestering specific endosomal vesicles in the perinuclear region, thereby establishing a homeostatic spatial architecture for the endosomal system [84, 85]. This tethering is crucial for coordinating subsequent events like vesicle fission, fusion, and sorting. To ensure the dynamic balance of membrane trafficking, the deubiquitinase ubiquitin‐specific protease 15 (USP15) plays a key antagonistic role. It can remove the ubiquitin chains from p62, allowing the tethered vesicles to be released for rapid transport to the cell periphery to fulfill their functions [84, 85]. This dynamic enzyme–counterenzyme regulation of p62's ubiquitination status by RNF26 and USP15 precisely controls the switching of vesicles between tethered and released states. This exquisite regulatory mechanism is indispensable for maintaining the homeostasis and function of membrane trafficking pathways such as endocytosis, secretion, and membrane protein recycling, further highlighting p62's central hub position in integrating PTMs signals to regulate organelle interactions and spatial organization.

### 2.7 Interplay and Integration of p62 PTMs in Cellular Homeostasis

Beyond the role of individual modifications in discrete pathways, the true regulatory power of the p62 PTMs network lies in the intricate interplay and integration of these modifications. This crosstalk ensures that cellular signals are not processed in isolation but are coordinated into a coherent and context‐appropriate functional output.

This interplay primarily manifests as a sequential cooperative model. The initiation of selective autophagy serves as a typical example: under energy or proteotoxic stress, ULK1 first phosphorylates S407 of p62, relieving UBA auto‐inhibition and creating a permissive state for subsequent S403 phosphorylation, which enhances ubiquitin substrate recognition, forming a “de‐repression‐activation” positive feedback loop that amplifies the signal [42, 43]. Another example is that T269/S272 phosphorylation promotes mTORC1 activation, and activated mTORC1 in turn phosphorylates S349, coupling anabolic metabolism with the antioxidant program [11, 76]. Furthermore, TRIM21‐mediated K63 ubiquitination at K7 inhibits p62 oligomerization [30]. This reduced oligomerization may in turn limit the access of upstream kinases such as mTORC1 to the S349 site, forming a feedback loop in which the modification state at one site (K7) regulates the modification efficiency at another site (S349) by controlling the interaction platform. Similarly, under oxidative stress, the formation of an intermolecular disulfide bond between C105 and C113 promotes p62 oligomerization, which in turn enhances its interaction with mTORC1 [50]. Since mTORC1 has been established as a kinase that phosphorylates p62 at S349 [11, 76], this enhanced interaction likely facilitates S349 phosphorylation, providing a direct mechanistic link between the cellular redox state and the activation of the Nrf2 antioxidant pathway. This intricate web of interactions ensures that the p62 PTMs network functions not as a collection of linear switches, but as a dynamic and integrated processor capable of interpreting complex cellular signals and orchestrating a harmonious, adaptive response.

Conversely, the network employs competitive or antagonistic regulation to act as a precise molecular switch, often at key hotspot residues. The lysine residue K420 is a quintessential example of this. Under nutrient deprivation, TIP60 acetylates K420 to promote phase separation and autophagy [2]. However, under distinct signals, the E3 ligase TRIM38 catalyzes a K63‐linked polyubiquitination at the very same K420 residue. This modification does not target p62 for degradation but instead creates a steric hindrance that physically blocks the p62–LC3 interaction, effectively shutting down autophagic flux [56]. Thus, acetylation and ubiquitination at K420 serve as mutually antagonistic signals, activation versus inhibition, directly competing to dictate p62's autophagic function. This principle extends to functional integration across different domains. While S349 phosphorylation is the key toggling on the Nrf2 antioxidant pathway [11], the overall amplitude of this response is gated by modifications controlling p62 protein stability, such as the antagonistic regulation of K7 ubiquitination by TRIM21 and USP13 [30, 62]. An effective antioxidant defense, therefore, requires not only the S349 on switch but also a sufficient pool of p62 protein, illustrating how modifications at distinct sites are functionally integrated.

## 3 The Central Role of p62 PTMs Network Dysregulation in Disease Pathogenesis

In contrast to the precise, dynamic, and reversible regulation observed under physiological conditions, specific PTMs of p62 often undergo persistent alterations in pathological contexts, such as sustained stress, aberrant oncogene activation, or dysfunction of key enzymes. This reprogrammed or locked‐in state of the PTMs network not only disrupts its normal regulatory functions but also transforms p62 from an adaptive signaling hub that maintains homeostasis into a core molecular switch that drives disease initiation and progression (Table 1).

**TABLE 1:** The role of p62 PTMs dysregulation in disease pathogenesis.

Disease category	Specific disease/pathological state	Involved PTMs site/type	Dysregulation mechanism/functional change	Pathological consequence	References
Cancer	Multiple cancers (pan‐cancer)	S349 phosphorylation	Persistent activation of p62 S349 phosphorylation, turning it into a high‐affinity decoy for Keap1, leading to sustained Nrf2 activation	Drives expression of antioxidant and prosurvival genes, enabling tumor cells to adapt to high‐ROS microenvironments, promoting tumorigenesis and progression	[11, 86, 87, 88, 89, 90, 91, 92]
	Breast cancer	S349 phosphorylation	VPS34 promotes PKC‐δ interaction with p62, enhancing S349 phosphorylation	Activates the pS349‐p62–Keap1–Nrf2 axis, promoting tumor progression	[86]
	Endometrial cancer	S349 phosphorylation	High glucose induces S349 phosphorylation via the mTOR–CK1–TAK1–PKC pathway	Phosphorylated p62 simultaneously releases both Nrf2 and LSD1 from Keap1, cooperatively driving pro‐oncogenic gene expression	[88]
	Hepatocellular carcinoma (HCV‐positive)	S349 phosphorylation	Drives Nrf2‐dependent metabolic reprogramming	Diverts glucose and glutamine to glutathione synthesis and the glucuronate pathway, conferring chemoresistance and proliferation advantages	[92]
	Multiple cancers (nutrient signaling)	T269/S272 phosphorylation	MEKK3–p38δ cascade‐mediated phosphorylation of p62 at T269/S272 (in response to nutrient signals) enables p62 to recruit TRAF6 to the lysosomal membrane and activate mTORC1	Promotes protein synthesis and cell growth in cancers such as prostate cancer	[76]
	Melanoma	T269 phosphorylation	DYRK3 kinase phosphorylates p62 specifically at T269 (but not S272), enhancing its interaction with TRAF6 and activating mTORC1 signaling	Promotes melanoma cell growth and tumor progression	[77]
	Multiple cancers (cell cycle regulation)	T269/S272 phosphorylation	Cdk1‐mediated phosphorylation of p62 at T269/S272 during mitosis stabilizes cyclin B1 and ensures normal cell cycle progression	Dysregulation of this phosphorylation leads to accelerated cell cycle progression, promoting proliferation and transformation in cancers such as lung cancer and osteosarcoma	[93, 94]
	Hepatocellular carcinoma	S272 phosphorylation	ATP6V1G1 overexpression upregulates p62 S272 phosphorylation	Drives tumor progression	[95]
	Renal cell carcinoma	Deubiquitination (stability)	HOXA3 transcriptionally activates USP15, which deubiquitinates and stabilizes the p62 protein	p62 accumulation inhibits autophagic flux and promotes CCL2 secretion, driving M2 polarization of tumor‐associated macrophages and accelerating tumor progression	[96]
	Breast cancer	K420 polyubiquitination (K63‐linked)	TRIM38 catalyzes K63‐linked ubiquitination, creating steric hindrance	Directly blocks p62 binding to LC3, inhibiting autophagic flux and exerting a tumor‐suppressive effect	[56]
	Prostate cancer bone metastasis	K63 ubiquitination (degradation)	SERPINH1 binds to p62, inhibiting TRIM21‐mediated K63 ubiquitin‐dependent degradation, thereby stabilizing p62	p62 inhibits apoptosis and promotes proliferation via the p62–Caspase 8 axis, driving bone metastasis	[97]
	Hepatocellular carcinoma	S28 phosphorylation	KHK‐A phosphorylates S28 under oxidative stress	Activates the Nrf2 pathway, promoting HCC development	[73]
	Hepatocellular carcinoma	K295 deacetylation (stability)	Sirt1‐mediated deacetylation stabilizes the p62 protein	Enhances downstream oncogenic signaling such as Nrf2, promoting hepatocarcinogenesis	[98]
	Chemotherapy/proteasome inhibitor resistance	S349 phosphorylation	Sustained activation of the Keap1–Nrf2 axis driven by p62 S349 phosphorylation upregulates drug efflux pumps and detoxifying enzymes	Mediates resistance to cisplatin (esophageal SCC, HCC) and proteasome inhibitors (multiple myeloma)	[99, 100, 101]
	Chemotherapy/proteasome inhibitor resistance	Dynamic switch: T269/S272 and S403 phosphorylation	Initial stress: p38δ‐mediated T269/S272 phosphorylation inhibits S403 phosphorylation, driving p62 to form protective aggregates; defective autophagosome closure: TBK1 hyperactivates S403 phosphorylation, forming nondegradable aggregates; PLK2‐mediated S349 phosphorylation solidifies aggregates	Enhances tolerance to proteotoxicity by forming aggregates of different states for emergency sequestration or physical containment, promoting drug resistance	[57, 58, 59, 102, 103]
	Head and neck cancer	S403 phosphorylation	Low MUL1 expression leads to persistent TBK1 activation, hyperphosphorylating p62 S403	Maintains autophagic flux to clear toxic protein aggregates generated by chemotherapeutics like cisplatin, promoting resistance	[104]
	Nasopharyngeal carcinoma	Stability (ubiquitination)	NAP1L1 stabilizes USP14, inhibiting ubiquitin‐mediated degradation of p62, leading to abnormal p62 accumulation and impaired autophagic flux	Induces resistance to cisplatin	[105]
	Radiotherapy resistance	S349 phosphorylation	Radiotherapy activates mTORC1‐mediated p62 S349 phosphorylation, weakening its binding to the immunosuppressive protein PROCR, leading to PROCR accumulation and IL‐6 secretion	Suppresses CD8^+^ T cell function, creating an immunosuppressive microenvironment	[106]
	Triple‐negative breast cancer	K420 polyubiquitination (K29/K63‐linked)	HECTD3 catalyzes K420 ubiquitination, promoting autophagic degradation of p62, reducing nuclear p62 levels and relieving inhibition of the DNA repair factor RNF168	Enhances homologous recombination repair efficiency, leading to radiotherapy resistance	[107]
	Immunotherapy resistance	S403/S349 phosphorylation	Tumor cells utilize TBK1‐ or CK1‐mediated p62 phosphorylation to promote autophagic degradation of the key immune signaling protein STING	Helps tumor cells evade immune surveillance, leading to immunotherapy resistance	[80, 81]
Neurodegenerative diseases	ALS/FTLD (TDP‐43 proteinopathy)	S403 phosphorylation	Loss‐of‐function TBK1 mutations (e.g., R573G) cause insufficient S403 phosphorylation; or PTK2/FAK–TBK1 axis drives excessive/persistent S403 phosphorylation	Insufficient phosphorylation leads to autophagy impairment and TDP‐43 accumulation; excessive phosphorylation forms hyperstable aggregates, obstructing autophagy and exacerbating neurotoxicity	[28, 108, 109, 110]
	ALS/FTLD	S349 phosphorylation	Disease‐associated p62 G427R mutation disrupts S349 phosphorylation	Impairs activation of the Keap1–Nrf2 antioxidant pathway, weakening neuronal capacity to clear ROS, accelerating neurodegeneration	[111]
	ALS	K420/K435 ubiquitination	Reduced NS1‐BP levels relieve inhibition of p62 ubiquitination, leading to increased p62 ubiquitination and degradation	Disrupts normal assembly and disassembly dynamics of stress granules, potentially exacerbating TDP‐43 pathology	[112]
	Parkinson's disease (PD)	S28 phosphorylation	PINK1‐mediated S28 phosphorylation (early stress)	Enhances p62's ubiquitin‐binding capacity, promoting autophagic clearance of toxic aggregates, exerting compensatory protection	[113]
	Parkinson's disease (PD)	K13 polyubiquitination (K48‐linked)	Pathogenic Parkin truncation variants lose the ability to ubiquitinate p62 at K13	Leads to abnormal p62 accumulation in neurons, resetting the threshold for protein degradation, accelerating neurodegeneration	[114, 115]
	Parkinson's disease (PD)	S349/S403 phosphorylation (inhibition)	LRRK2 kinase (especially G2019S mutant) inhibits p62 S349 and S403 phosphorylation	Hinders p62 activation of the Keap1–Nrf2 antioxidant signaling pathway	[116]
	Parkinson's disease (PD)	T138 phosphorylation	LRRK2‐mediated T138 phosphorylation	May promote neuronal apoptosis by impairing p62's ability to activate the Nrf2 pathway	[74]
	Parkinson's disease (PD)/Lewy body dementia	C331 S‐nitrosylation	Pathological levels of NO lead to S‐nitrosylation at C331	Enhances p62 binding to LC3 but blocks autophagosome–lysosome fusion, causing autophagic flux arrest and promoting α‐synuclein secretion via extracellular vesicles	[33]
	Alzheimer's disease (AD)	S349 phosphorylation	Significantly elevated and enriched within neurofibrillary tangles (NFTs)	Marks comprehensive collapse of proteostasis and a failed attempt at antioxidant response	[117]
	Alzheimer's disease (AD)	C44 S‐nitrosylation	Pathological NO‐mediated S‐nitrosylation within the PB1 domain	May impair p62's core functions in selective autophagy and signal transduction by interfering with its oligomerization or scaffolding function	[118]
	Alzheimer's disease (AD)	Functional decoupling	The link between p62 S349 phosphorylation and Keap1 binding/downstream Nrf2 activation is severed	Neurons lose the ability to initiate adaptive antioxidant defense via the p62–Keap1–Nrf2 axis, becoming vulnerable to oxidative damage	[119]
	Huntington's disease (HD)	C289/C290 S‐acylation (palmitoylation) loss	S‐acylation levels of p62 are significantly reduced	p62 loses normal membrane localization, failing to effectively deliver mutant huntingtin (mHTT) to the autophagic degradation pathway, leading to toxic protein aggregation	[120]
	Multiple sclerosis (MS)	K314 ubiquitination	RNF6‐mediated ubiquitination at K314	Reshapes p62 function in endothelial cells, causing hyperactive autophagy that consumes blood–spinal cord barrier junctional proteins, compromising barrier integrity and promoting immune cell infiltration	[121]
Metabolic diseases	Type 2 diabetes	T269/S272 phosphorylation	Gut microbial metabolite imidazole propionate activates p38γ MAPK, phosphorylating p62 T269/S272	Aberrantly activates mTORC1 signaling, inhibiting insulin signaling, contributing to insulin resistance	[122]
	MASLD to MASH progression	S403 phosphorylation	Early: cGAS–STING–TBK1 axis activation promotes S403 phosphorylation, clearing toxic aggregates (compensatory); Late: TBK1 activity declines, S403 phosphorylation insufficient	Early protective compensation; late insufficient S403 phosphorylation impairs mitophagy and other protective mechanisms, exacerbating liver injury	[123, 124, 125]
	Obesity‐related cardiomyopathy	K63 polyubiquitination	Downregulation of deubiquitinase USP25 leads to aberrant K63 ubiquitination of p62	Impairs p62's autophagy receptor function, preventing effective recognition and degradation of proinflammatory kinase TAK1, driving cardiomyocyte hypertrophy and fibrosis	[126]
Infectious diseases	Viral (e.g., DNA virus) infection	S403 phosphorylation	Host mediates S403 phosphorylation via the TRIM23–TBK1 axis	Promotes degradation of viral components via selective autophagy (host defense)	[79]
	Viral infection (immune negative regulation)	K7 deubiquitination (K63‐linked)	OTUD7B mediates K63‐linked deubiquitination at K7	Promotes p62 oligomerization, accelerating autophagic degradation of transcription factor IRF3, preventing excessive interferon signaling	[127]
	Hepatitis B virus (HBV) infection	S403 phosphorylation	HBV surface antigen hijacks TBK1, altering its substrate preference to catalyze p62 S403 phosphorylation	Creates an autophagy‐favorable environment for viral replication and blocks autophagosome–lysosome fusion, causing autophagic flux arrest	[128]
	Coronavirus infection	T138 phosphorylation	Coronavirus M protein activates host PDPK1 kinase to catalyze p62 T138 phosphorylation	Inhibits antiviral xenophagy, instead aberrantly activating mitophagy to clear mitochondrial immune signaling platforms	[129]
	Listeria monocytogenes infection	S407 phosphorylation (inhibition)	Bacterial ActA protein blocks ULK1‐mediated p62 S407 phosphorylation	Evades xenophagic clearance by host cells	[130]
	Salmonella infection	S351 phosphorylation (inhibition)	Bacteria inhibit AKT–mTOR signaling via NLRP6, reducing p62 S351 phosphorylation levels	Weakens the host's Nrf2 antioxidant defense pathway	[131]
	Trichinella spiralis infection	K422 polyubiquitination (K63‐linked)	Parasite‐secreted E3 ligase Ts‐RNF catalyzes K63‐linked ubiquitination of host p62 at K422	Weakens p62‐mediated mitophagy, interfering with host myocyte differentiation and favoring parasite survival	[132]
Inflammatory and autoimmune diseases	Systemic lupus erythematosus (SLE)	K63 polyubiquitination	TRIM21 catalyzes K63 ubiquitination of p62	Blocks normal binding of p62 to STING, causing abnormal STING accumulation and driving persistent Type I interferon pathway activation	[133]
	Acute lung injury	K165 deacetylation	HDAC10 mediates deacetylation of p62 at K165 in macrophages	Hyper‐stabilizes p62 protein and aberrantly activates autophagic flux, exacerbating tissue damage	[134]
	Osteoarthritis	Upregulated expression (indirect)	IL‐1β upregulates p62 expression via the MSK1–NF‐κB p65 pathway	p62‐promoted protective mitophagy is a compensatory mechanism in stressed chondrocytes; failure of this mechanism accelerates cartilage degeneration	[135]
Muscle‐related diseases	Cancer cachexia	S351 phosphorylation	IL‐1β–NBR1 axis activates p62 S351 phosphorylation, driving Nrf2‐mediated antioxidant enzyme expression (early compensation)	Early protective response against catabolism; during progression, this pathway becomes insufficient or fails to activate, accelerating muscle atrophy	[136, 137]
Ischemic injury	Ischemic stroke/reperfusion injury	S403 phosphorylation (insufficient)	Parkin–TBK1–p62 signaling axis fails to activate adequately, leading to insufficient p62 S403 phosphorylation	Unable to effectively initiate mitophagy, damaged mitochondria accumulate, exacerbating oxidative stress and cell death	[138]
	Hypoxic–ischemic brain injury	Stability (ubiquitination)	Exosomal miR‐653‐3p inhibits TRIM21, blocking ubiquitin‐mediated degradation of p62, stabilizing the protein	Stabilized p62 activates the Nrf2 antioxidant pathway and downstream anti‐inflammatory programs, exerting a protective effect	[139]
Cardiac aging	Chronic stress and cardiac aging	Dynamic evolution: S349/S403 phosphorylation	Early: p62 and its phosphorylation levels increase, autophagy enhanced, maintaining high cardiac function (compensation); Late: autophagic flux impaired, p62 and LC3 form abnormal insoluble inclusions	p62 transitions from adaptive protection to decompensatory collapse; its aberrant phosphorylation is both a manifestation of impaired autophagic flux and a driver of a vicious cycle, leading to dilated cardiomyopathy and heart failure	[140]
Fibrotic diseases	Renal fibrosis	K63 polyubiquitination	TRIM21 expression is upregulated, catalyzing K63 ubiquitination of p62	Inhibits p62 oligomerization, impairing its core function as an autophagy receptor, preventing effective autophagic clearance of profibrotic TGF‐β1, leading to TGF‐β1 accumulation and driving fibrosis	[141]

Abbreviations: S349 refers to Serine 349; ROS, reactive oxygen species; VPS34, vacuolar protein sorting 34; PKC‐δ, protein kinase C delta; mTOR, mechanistic target of rapamycin; CK1, casein kinase 1; TAK1, transforming growth factor‐β‐activated kinase 1; LSD1, lysine‐specific demethylase 1; Nrf2, nuclear factor erythroid 2‐related factor 2; T269, Threonine 269; S272, Serine 272; MEKK3, MAP/ERK kinase kinase 3; TRAF6, TNF receptor‐associated factor 6; mTORC1, mechanistic target of rapamycin complex 1; DYRK3, dual‐specificity tyrosine phosphorylation‐regulated kinase 3; Cdk1, cyclin‐dependent kinase 1; ATP6V1G1, ATPase H+ transporting V1 subunit G1; HOXA3, homeobox A3; USP15, ubiquitin‐specific protease 15; CCL2, C–C motif chemokine ligand 2; K420, Lysine 420; LC3, microtubule‐associated protein 1 light chain 3; TRIM38, tripartite motif‐containing protein 38; SERPINH1, serpin family H member 1; TRIM21, tripartite motif‐containing protein 21; S28, Serine 28; KHK‐A, ketohexokinase isoform A; K295, Lysine 295; Sirt1, Sirtuin 1; EGFR, epidermal growth factor receptor; Y433, Tyrosine 433; S403, Serine 403; p38δ, p38 delta mitogen‐activated protein kinase; PLK2, polo‐like kinase 2; TBK1, TANK‐binding kinase 1; MUL1, mitochondrial E3 ubiquitin protein ligase 1; NAP1L1, nucleosome assembly protein 1‐like 1; USP14, ubiquitin‐specific protease 14; PROCR, protein C receptor; IL‐6, interleukin‐6; HECTD3, HECT domain E3 ubiquitin protein ligase 3; RNF168, ring finger protein 168; STING, stimulator of interferon genes; ALS, amyotrophic lateral sclerosis; FTLD, frontotemporal lobar degeneration; TDP‐43, TAR DNA‐binding protein 43; PTK2, protein tyrosine kinase 2; FAK, focal adhesion kinase; OPTN, optineurin; G427R, Glycine 427 to arginine mutation; NS1‐BP, NS1‐binding protein; PD, Parkinson's disease; PINK1, PTEN‐induced kinase 1; K13, Lysine 13; LRRK2, leucine‐rich repeat kinase 2; T138, Threonine 138; NO, nitric oxide; C331, Cysteine 331; AD, Alzheimer's disease; NFTs, neurofibrillary tangles; C44, Cysteine 44; HD, Huntington's disease; C289, Cysteine 289; C290, Cysteine 290; MS, multiple sclerosis; RNF6, ring finger protein 6; K314, Lysine 314; AMPK, AMP‐activated protein kinase; cGAS, cyclic GMP–AMP synthase; MASH, metabolic dysfunction‐associated steatohepatitis; MASLD, metabolic dysfunction‐associated steatotic liver disease; USP25, ubiquitin‐specific protease 25; OTUD7B, OTU domain‐containing protein 7B; IRF3, interferon regulatory factor 3; HBV, hepatitis B virus; PDPK1, 3‐phosphoinositide‐dependent protein kinase 1; K422, Lysine 422; SLE, systemic lupus erythematosus; HDAC10, histone deacetylase 10; K165, Lysine 165; IL‐1β, interleukin‐1 beta; MSK1, mitogen‐ and stress‐activated protein kinase 1; NF‐κB, nuclear factor kappa‐light‐chain‐enhancer of activated B cells; S351, Serine 351; S405, Serine 405; TGF‐β1, transforming growth factor‐beta 1; ESCRT, endosomal sorting complexes required for transport.

### 3.1 Cancer

p62 is significantly overexpressed in a variety of malignant tumors. The dysregulation of its PTMs network has transcended a mere correlative phenomenon to become a core mechanism driving malignant tumor progression and therapy resistance. This network constitutes a crucial molecular foundation linking oncogenic signaling, metabolic reprogramming, immune microenvironment remodeling, and drug resistance, exhibiting complex and highly context‐dependent functions.

#### 3.1.1 Driving Tumorigenesis and Malignant Progression: Oncogenic Reprogramming of the p62 PTMs Network

The persistent activation of specific p62 PTMs directly drives the initiation and progression of various tumors by sustaining core prosurvival and proliferation signaling pathways.

The widespread oncogenic role of the p‐S349 p62–Keap1–Nrf2 axis is the most prominent mechanism within this network. Its core lies in this modification transforming p62 into a high‐affinity decoy for Keap1. By competitively inhibiting Keap1‐mediated degradation of Nrf2, it leads to stable nuclear translocation of Nrf2, persistently driving the expression of antioxidant and prosurvival genes, thereby enabling tumor cells to adapt to high‐ROS microenvironments [11]. In breast cancer, vacuolar protein sorting 34 (VPS34) promotes the dissociation of Nrf2 from the Keap1–Nrf2 complex by competitively binding to Keap1, facilitating Nrf2's entry into the nucleus and subsequently activating Nrf2‐mediated p62 transcription. At the same time, VPS34 also enhances the interaction between PKC‐δ and p62, promoting the phosphorylation of p62 at S349, which further activates the p‐S349 p62–Keap1–Nrf2 signaling pathway and ultimately promotes tumor progression [86]. Long‐term metabolic pressure in conditions like glycogen storage disease Type 1a leads to murine p62 phosphorylation at S351 (corresponding to human S349) via the phosphoinositide 3‐kinase (PI3K)/v‑akt murine thymoma viral oncogene homolog (Akt)/mTOR pathway, which in turn drives liver tumorigenesis in mice [87]. In endometrial cancer, high glucose induces this modification by activating the mTOR–CK1–TAK1–protein kinase C (PKC) pathway. Phosphorylated p62 can simultaneously liberate both Keap1 substrates, Nrf2 and lysine‐specific demethylase 1 (LSD1), cooperatively driving pro‐oncogenic gene expression [88]. Estrogen signaling converges on p62 S349 phosphorylation via the Src–EGFR–PI3K–Akt–mTOR axis, causing the release of estrogen receptor α bound to Keap1, providing dual impetus for estrogen‐driven endometrial cancer growth [89]. In glioblastoma, aberrant Src kinase, by phosphorylating and reprogramming Caspase 8 function, subsequently activates mTORC1 and drives p62 S349 phosphorylation, leading to sustained Nrf2 signaling and metabolic reprogramming [90]. This axis is also exploited by viruses. Kaposi's sarcoma‐associated herpesvirus infection induces p62 S349 phosphorylation, persistently activating the Keap1–Nrf2 axis to create a favorable microenvironment for viral latency and potential tumorigenesis [91]. In hepatitis C virus (HCV)‐positive hepatocellular carcinoma, this axis drives Nrf2‐dependent metabolic reprogramming, diverting glucose and glutamine to glutathione synthesis and the glucuronate pathway, which confers chemoresistance and proliferation advantages [92].

T269/S272 phosphorylation is another key PTMs event closely associated with tumor growth and cell cycle progression. Under nutrient signaling, MEKK3–p38δ cascade‐mediated phosphorylation of p62 at T269/S272 enables p62 to act as a scaffold, recruiting TRAF6 near the lysosomal membrane to subsequently activate mTORC1, promoting protein synthesis and cell growth. This mechanism is prevalent in prostate cancer and melanoma [76, 77]. Overexpression of the V‐ATPase subunit ATPase H+ transporting V1 subunit G1 (ATP6V1G1) in hepatocellular carcinoma can drive tumor progression by upregulating p62 S272 phosphorylation [95]. Furthermore, this phosphorylation event directly participates in cell cycle regulation. In lung cancer or osteosarcoma, cyclin‐dependent kinase 1 (Cdk1)‐mediated T269/S272 phosphorylation ensures normal mitosis by stabilizing cyclin B1. Loss of this function, conversely, leads to accelerated cell cycle progression, directly promoting proliferation and transformation [93, 94].

Beyond the core phosphorylation modifications described above, other p62 PTMs events also profoundly influence tumor progression through various mechanisms such as regulating autophagy and the immune microenvironment, demonstrating potential as therapeutic targets. In renal cell carcinoma, the transcription factor homeobox A3 (HOXA3) transcriptionally activates the deubiquitinase USP15, which deubiquitinates and stabilizes p62. Accumulated p62 not only inhibits autophagic flux but also positively regulates the secretion of the chemokine C‐C motif chemokine ligand 2 (CCL2), driving tumor‐associated macrophages toward tumor promoting M2 polarization, thereby accelerating tumor progression [96]. Conversely, in breast cancer, the E3 ligase TRIM38 catalyzes a unique nondegradative K63‐linked polyubiquitination of p62 at K420, which directly creates steric hindrance to block p62's binding to LC3, thereby inhibiting autophagic flux and exerting a tumor‐suppressive effect [56]. In prostate cancer bone metastasis, the ER chaperone serpin family H member 1 (SERPINH1) binds to p62, inhibiting its TRIM21‐mediated K63 ubiquitin‐dependent degradation, thus stabilizing p62. This stabilization, in turn, inhibits apoptosis and promotes proliferation via the p62–Caspase 8 axis, driving bone metastasis [97]. In lung adenocarcinoma cells under glucose starvation, glutamate dehydrogenase 1 (GLUD1) is deacetylated by sirtuin 3 (SIRT3) and localized to the cytosol. By binding to histone deacetylase 6 (HDAC6), it promotes p62 deacetylation, thereby inhibiting p62 body formation and autophagic cell death, aiding tumor cell survival [142]. Additionally, S28 phosphorylation of p62 by KHK‐A under oxidative stress and Lysine 295 (K295) deacetylation mediated by Sirt1 can both block p62 ubiquitination dependent proteasome degradation, thereby independently activating downstream Nrf2 signaling and promoting hepatocellular carcinoma development [73, 98].

#### 3.1.2 Orchestrating Multimodal Therapeutic Resistance: Mechanistic Integration by the p62 PTMs Network

Through its PTMs network, p62 mediates resistance to various cancer therapies, with mechanisms that vary depending on the treatment modality.

In the context of chemotherapy and proteasome inhibitor therapy, p62, through its intricate PTMs network in either persistently activated or dynamically switching forms, is deeply involved in the resistance mechanisms of various diseases. First, sustained activation of the Keap1–Nrf2 axis driven by p62 S349 phosphorylation is a common mechanism mediating classical drug resistance. This pathway reduces intracellular drug concentrations by upregulating drug efflux pumps and detoxification enzymes, thereby mediating resistance to cisplatin in cancers such as esophageal squamous cell carcinoma and hepatocellular carcinoma [100, 101]. Abnormalities in upstream regulators can further strengthen this axis. For instance, in multiple myeloma, tripartite motif‐containing protein 44 (TRIM44) drives persistent Nrf2 activation by promoting p62 oligomerization and S349 phosphorylation, leading to resistance to proteasome inhibitors [99]. Second, under proteotoxic stress induced by proteasome inhibitors, p62's function dynamically switches, forming aggregates of different states to create a multilayered defense that promotes resistance. During the initial stress, T269/S272 phosphorylation mediated by p38 delta (p38δ) can inhibit its proautophagic S403 phosphorylation, driving p62 to encapsulate ubiquitinated proteins into protective aggregates (aggresomes), achieving emergency sequestration to aid cell survival [57, 58, 103]. If the autophagosome closure mechanism is defective (e.g., due to vacuolar protein sorting‐associated protein 37A (VPS37A) deficiency), accumulated unclosed phagophores abnormally activate TBK1, which strongly phosphorylates p62 S403, also driving the formation of nondegradable protective aggregates [102]. As stress persists, the specifically induced kinase PLK2 promotes the solidification and condensation of dynamic aggregates into large, insoluble structures through p‐S349 p62, laying the groundwork for subsequent sequestration or clearance [59]. These p62‐mediated, sequential or parallel dynamic transformations of aggregates collectively enhance cellular tolerance to proteotoxicity induced by proteasome inhibitors. Furthermore, in specific tumor types, p62 also constitutes a core defense through other modification axes. For example, in head and neck cancer, low expression of mitochondrial E3 ubiquitin protein ligase 1 (MUL1) impedes the degradation of active TBK1, leading to overactivation of the TBK1–p62 S403 axis. This promotes resistance by maintaining autophagic flux to clear toxic protein aggregates generated by chemotherapeutic drugs like cisplatin. Using TBK1 inhibitors can block this axis and sensitize cells to cisplatin [104]. In nasopharyngeal carcinoma, the nucleosome assembly protein 1‐like 1 (NAP1L1) stabilizes the deubiquitinase ubiquitin‐specific protease 14 (USP14), thereby inhibiting the ubiquitin‐mediated degradation of p62. This leads to abnormal p62 accumulation and impedes autophagic flux, inducing resistance to cisplatin. Conversely, the E3 ubiquitin ligase F‐box/WD repeat‐containing protein 7 (FBXW7) mediates the ubiquitination and degradation of NAP1L1, subsequently relieving its stabilizing effect on p62, restoring autophagic function, and ultimately resensitizing tumor cells to chemotherapy [105].

In radiotherapy resistance, p62 modifications play an important role by affecting the immune microenvironment or DNA repair. Radiotherapy can activate mTORC1‐mediated phosphorylation of p62 S349. This modification specifically weakens the binding of p62 to the protein C receptor (PROCR), leading to PROCR accumulation and driving interleukin‐6 (IL‐6) secretion, which suppresses CD8^+^ T cell function, thereby creating an immunosuppressive microenvironment [106]. Concurrently, in triple‐negative breast cancer, the E3 ligase HECT domain‐containing E3 ubiquitin protein ligase 3 (HECTD3) catalyzes K29/K63‐linked polyubiquitination of p62 at K420, promoting its autophagic degradation. The reduction of nuclear p62 relieves its inhibition of the DNA repair key factor RING finger protein 168 (RNF168), thereby enhancing homologous recombination repair efficiency, leading to radiotherapy resistance. Thus, inhibiting HECTD3 can radiosensitize the cells [107].

Regarding targeted and immunotherapy, the role of p62 exhibits context and cell type specificity. For example, in glioblastoma targeted therapy, proviral integration site for Moloney murine leukemia virus (PIM) kinase‐mediated phosphorylation of p62 Serine 332 (S332) can promote the degradation of the death receptor TNF‐related apoptosis‐inducing ligand receptor 2/death receptor 5 (TRAIL‐R2/DR5) via autophagy or accelerate its endocytosis, thereby attenuating the apoptotic signaling induced by TRAIL therapy [143]. In immunotherapy, p62's function demonstrates dual and opposing regulatory roles. On one hand, within CD8^+^ tumor‐infiltrating T lymphocytes, metformin can activate selective autophagy through an Nrf2‐mediated metabolic feedback circuit, subsequently promoting glutaminolysis and activating mTORC1. The activated mTORC1, in turn, phosphorylates murine p62 S351, forming a positive feedback loop to persistently enhance Nrf2 signaling. This pathway is crucial for maintaining the proliferation and effector function of CD8^+^ T lymphocytes and can synergize with anti‐programmed cell death protein 1 (PD‐1) antibody therapy [144]. On the other hand, tumor cells may utilize TBK1‐ or CK1‐mediated phosphorylation of p62 (at S403 or S349) to promote the autophagic degradation of the key immune signaling protein STING. This mechanism potentially helps tumor cells evade immune surveillance, leading to immunotherapy resistance [80, 81].

In summary, through its complex, dynamic, and highly context‐dependent PTMs network, p62 not only drives tumorigenesis but also endows tumor cells with a powerful adaptive capacity to withstand various therapeutic pressures. From directly activating prosurvival pathways to flexibly switching between degradation and sequestration functions and to modulating the immune microenvironment and DNA repair, p62 has become a core molecular target for understanding tumor drug resistance and developing novel combination therapeutic strategies.

### 3.2 Neurodegenerative Diseases

In neurodegenerative diseases such as Alzheimer's disease (AD), Parkinson's disease (PD), and amyotrophic lateral sclerosis/frontotemporal dementia (ALS/FTLD), aberrant aggregation of misfolded proteins and collapse of neuronal homeostasis are central pathological features. p62, as a key receptor for selective autophagy and an integrator of stress signals, sees its normal PTMs network profoundly dysregulated, thereby critically influencing the fate of pathological protein aggregates and neuronal viability. This section will systematically elucidate, based on different disease contexts, how the disruption of p62 PTMs drives specific pathological processes.

In ALS/FTLD, particularly in TAR DNA‐binding protein 43 (TDP‐43) proteinopathies, the functional balance of the TBK1–p62 S403 phosphorylation axis is crucial. Physiological levels of S403 phosphorylation are necessary for clearing toxic proteins, and loss of this function (e.g., due to the TBK1 R573G mutation) leads to autophagic impairment and TDP‐43 accumulation [28, 109, 110]. However, under sustained pathological conditions, excessive or persistent S403 phosphorylation driven by the focal adhesion kinase (FAK)–TBK1 axis paradoxically inhibits p62‐mediated clearance of insoluble aggregates, which may be due to the formation of hyperstable aggregates that physically obstructs autophagy and the premature degradation of functional p62 via dysregulated ubiquitination, thereby exacerbating neurotoxicity [108]. Human genetic evidence reinforces the centrality of this axis: TBK1 is the second most common causative gene for FTD, and mutations in its interacting protein optineurin (OPTN) also impair kinase activity or autophagosome recruitment [145]. Furthermore, the p62–Keap1–Nrf2 antioxidant defense axis is significantly compromised in these diseases. The disease‐associated p62 G427R mutation disrupts its S349 phosphorylation, impairing the activation of the Keap1–Nrf2 pathway. This renders neurons unable to mount an effective antioxidant gene expression program, severely weakening their capacity to clear ROS, thereby accelerating neurodegeneration [111]. Additionally, p62 PTMs dysregulation extends to the homeostasis of membraneless organelles like stress granules. In ALS, reduced levels of the adaptor protein NS1‐binding protein (NS1‐BP) attenuate its inhibitory effect on the ubiquitination status of p62 at K420 and K435. The aberrantly increased ubiquitination of p62 promotes its own degradation, disrupting the normal assembly and disassembly dynamics of stress granules, a process in which p62 participates [112]. This not only affects the cell's immediate stress response but may also indirectly exacerbate the pathological conversion of TDP‐43 by disturbing RNA‐protein homeostasis. It should be noted that p62's role in stress granule dynamics and the precise regulatory mechanisms of its ubiquitination remain active areas of investigation.

In PD, dysregulation of the p62 PTMs network is a core element driving multifaceted pathology. Different p62 PTMs collectively lead to neuronal homeostasis collapse and degeneration by interfering with key pathways such as protein clearance and antioxidant defense. The temporal dysregulation of p62 PTMs is particularly critical in protein clearance. During early stress, PTEN‐induced putative kinase 1 (PINK1)‐mediated phosphorylation of S28 enhances p62's ubiquitin‐binding capacity, promoting the autophagic clearance of toxic aggregates and exerting compensatory protection [113]. However, long‐term proteostasis imbalance is directly linked to Parkin‐mediated K48‐linked polyubiquitination at Lysine 13 (K13), a key mechanism regulating p62 degradation via the proteasome. Pathogenic Parkin truncation variants identified in patients (e.g., N52Mfs29 and L358Rfs77) lose this ubiquitination capability, leading to abnormal accumulation of p62 in neurons. While this accumulation might initially enhance autophagy, prolonged imbalance resets the threshold for protein degradation, impairing the neuron's ability to cope with sustained stress and thus accelerating degeneration [114, 115]. Regarding antioxidant defense, PTMs abnormalities directly weaken cellular self‐protection. The LRRK2 kinase (especially the G2019S mutant) can inhibit phosphorylation of p62 at S349 and S403, thereby hindering its activation of the Keap1–Nrf2 antioxidant signaling pathway [116]. Furthermore, LRRK2 can phosphorylate p62 at T138, which may promote neuronal apoptosis by impairing its ability to activate the Nrf2 pathway [74]. Crucially, S‐nitrosylation, a modification derived from oxidative stress, directly bridges impaired autophagy and pathological protein propagation. In PD, pathological levels of nitric oxide (NO) lead to S‐nitrosylation of p62 at Cysteine 331 (C331). This aberrant modification enhances p62 binding to LC3 but blocks autophagosome–lysosome fusion, causing autophagic flux arrest. The direct pathological consequence is that pathological α‐synuclein within undegraded autophagosomes is instead secreted in large quantities via extracellular vesicles, thereby promoting the interneuronal spread of pathological proteins and driving disease dissemination and progression [33].

In the brains of AD patients, the phosphorylation level of p62 at S349 is significantly elevated and specifically enriched within neurofibrillary tangles (NFTs) composed of hyperphosphorylated Tau protein [117]. This phenomenon carries dual pathological significance. First, it marks the comprehensive collapse of the neuronal proteostasis system, where autophagic clearance capacity is insufficient to cope with abnormal protein aggregation. Second, it reflects a failed attempt by the cell to persistently activate antioxidant responses to counter sustained oxidative stress. Therefore, p‐S349 p62 is not only a biomarker for AD but also molecular evidence of the simultaneous disruption of the two major protective systems: protein clearance and antioxidant defense. Concurrently, p62 is a highly enriched target of S‐nitrosylation in the AD brain. The key modification site is Cysteine 44 (C44) within its PB1 domain [118]. This modification, mediated by pathological levels of NO, may directly impair p62's core functions in selective autophagy and signal transduction by interfering with its oligomerization capacity, thereby exacerbating neuronal dysfunction in the context of amyloid‐β (Aβ) and Tau pathology. More profoundly, the p62 PTMs network in AD exhibits a functional disconnect. Studies reveal that even when proteasome dysfunction in cortical neurons successfully induces enhanced p62 S349 phosphorylation, this phosphorylation fails to effectively drive its binding to Keap1 and subsequent Nrf2 activation [119]. This critical signal decoupling indicates that under AD pathology, the normal link between p62 PTMs (e.g., pS349) and their downstream protective functions (e.g., activating antioxidant genes) is severed. The consequence is that neurons not only lose the ability to initiate adaptive antioxidant defense via the p62–Keap1–Nrf2 axis but may also be trapped in a state of “signaling without protection,” making them vulnerable to oxidative damage and accelerating the degenerative process.

In the pathology of Huntington's disease (HD), dysregulation of the p62 PTMs network is also a key driver, with the loss of reversible S‐acylation (palmitoylation) being particularly prominent. This lipid modification occurs at Cysteines 289 and 290 (C289 and C290) of p62 and serves as a molecular navigation signal regulating its precise intracellular localization and function. Under physiological conditions, S‐acylation ensures p62 can correctly target and anchor to key membrane sites for autophagosome formation or lysosomal degradation, thereby efficiently mediating the recognition and loading of ubiquitinated toxic protein aggregates. However, in the brain of HD patients, the S‐acylation level of p62 is significantly reduced [120]. The loss of this PTM causes p62 to lose its normal membrane localization ability, preventing it from effectively delivering toxic aggregates like mutant huntingtin (mHTT) for autophagic degradation. The direct pathological consequence is the aberrant retention and accumulation of toxic protein aggregates in the cytoplasm, forming inclusions that are difficult to clear.

In pathological models of autoimmune demyelinating diseases like multiple sclerosis (MS), abnormalities in p62 PTMs directly participate in the initiation and amplification of central nervous system immune attacks, with the E3 ligase RING finger protein 6 (RNF6)‐mediated specific ubiquitination at Lysine 314 (K314) being a key pathogenic link. Under disease‐associated signaling, RNF6 is significantly activated and subsequently catalyzes the ubiquitination of p62 at K314 [121]. This PTM is not a simple metabolic tag but a pathogenic modification. It directly reshapes p62's function in endothelial cells, causing the autophagy process to shift from homeostasis maintenance to runaway activation. The excessively active autophagic flux abnormally consumes key structural and junctional proteins of blood–spinal cord barrier endothelial cells, compromising the barrier's tightness and integrity. This PTM‐driven barrier compromise has direct neuroimmunopathological consequences: peripheral immune cells (e.g., T cells, macrophages) that are normally strictly confined to the periphery can abnormally infiltrate into the central nervous system. Once inside, these activated immune cells trigger intense inflammatory responses and directly attack the myelin sheaths wrapping nerve axons, leading to demyelination, axonal injury, and neurological dysfunction, thereby driving disease progression.

Beyond directly affecting the homeostasis of mature neurons, p62 PTMs dysregulation also acts earlier at the source of neuroregeneration, fundamentally weakening the brain's repair and compensatory capacity. In neural stem cells, the autophagy‐initiating protein FIP200 acts as a key negative regulator of the TBK1–p62 S403 phosphorylation axis. When FIP200 function is lost, this regulatory axis becomes imbalanced: TBK1 is excessively activated, leading to aberrant persistent phosphorylation of p62 S403 and the formation of pathological aggregates [146]. The direct consequence is the impairment of neural stem cell self‐renewal and differentiation potential, preventing them from effectively replenishing damaged neuronal networks. Under pathological conditions such as energy stress, AMPK‐mediated phosphorylation of p62 at Serine 294 (S294) can be aberrantly triggered. This modification excessively promotes p62 translocation to mitochondria, triggering uncontrolled mitophagy. The pathological consequence lies in the fact that this excessive clearance may initiate autophagic cell death program, particularly in neural stem cells of critical brain regions like the hippocampus, directly depleting the cellular reservoir for neuroregeneration and repair [147].

In summary, in neurodegenerative diseases, the PTMs network of p62 undergoes a cascading collapse from fine imbalance to profound disruption. Its dysregulation is not the failure of a single link but manifests as abnormal persistence of specific modifications, paradoxical reversal of physiological functions, and fatal decoupling of upstream and downstream signals. These abnormalities collectively erode p62's multiple core functions, including clearing protein aggregates, maintaining antioxidant defense, executing organelle quality control, and safeguarding neuroregeneration.

### 3.3 The Role of the p62 PTMs Network in Metabolic Diseases

The dysregulation of the p62 PTMs network plays a crucial role in the pathogenesis and progression of various metabolic diseases, with mechanisms exhibiting tissue specificity and dynamic evolution.

In Type 2 diabetes, the gut microbial metabolite imidazole propionate, a histidine‐derived compound, activates the p38γ MAPK, leading to phosphorylation of p62 at T269/S272. This in turn aberrantly activates mTORC1 signaling, which inhibits insulin signaling by suppressing tyrosine phosphorylation of insulin receptor substrate and reducing its protein levels, ultimately contributing to insulin resistance and the onset of Type 2 diabetes [122].

During the progression from metabolic dysfunction‐associated steatotic liver disease (MASLD) to steatohepatitis (MASH), the p62 PTMs network undergoes dynamic changes. Studies show that in MASH livers, p62 undergoes significant phosphorylation at S403 and coaggregates with ubiquitinated proteins to form insoluble aggregates, constituting the core of Mallory‐Denk bodies, concurrently accompanied by impaired autophagic flux [123]. In the early stages of the disease, lipotoxic stress can activate the cGAS–STING–TBK1 axis, phosphorylating p62 S403 to promote the clearance of toxic aggregates, thereby exerting a compensatory effect. However, in advanced MASH accompanied by TBK1 functional exhaustion, decreased TBK1 kinase activity leads to the functional exhaustion of this axis, resulting in insufficient p62 S403 phosphorylation, which subsequently impairs protective mechanisms such as mitophagy, ultimately exacerbating liver injury [124, 125]. Notably, this stage contrasts with earlier observations of elevated p62 S403 phosphorylation‐positive aggregates in MASH livers, highlighting a dynamic transition from compensatory activation to terminal failure of the p62‐mediated quality control system, a temporal regulatory mechanism that warrants further investigation.

In the pathological process of obesity‐related cardiomyopathy, abnormal K63‐linked polyubiquitination of p62 is a key pathogenic event. Research has found that under conditions of obesity or lipotoxic stress, the expression of the deubiquitinase ubiquitin‐specific protease 25 (USP25) is downregulated in cardiomyocytes, leading to abnormal K63 ubiquitination of p62. This modification directly impairs the autophagic receptor function of p62, preventing it from effectively recognizing and degrading the key proinflammatory kinase TAK1. The aberrant accumulation of TAK1 then persistently activates downstream inflammatory signaling pathways such as MAPK, driving cardiomyocyte hypertrophy, fibrosis, and cardiac dysfunction. Mechanistically, USP25, via its active site Cysteine 178 (C178), directly binds to and removes K63 ubiquitin chains from p62. This deubiquitination process is crucial for maintaining the normal function of p62 [126].

In summary, through the precise regulation of its PTMs such as phosphorylation and ubiquitination, p62 is deeply involved in adaptive responses to metabolic stress. The imbalance of its network serves as an important molecular hub connecting nutrient excess, insulin resistance, organ inflammation, and fibrosis.

### 3.4 The Role of the p62 PTMs Network in Infectious Diseases

The PTMs network of p62 occupies a central position in host anti‐infective immunity, constituting a dynamic offensive and defensive hub. Its mechanisms are reflected in the host's active utilization of p62 PTMs to clear pathogens, as well as the pathogen's reverse hijacking of this network to achieve immune evasion, both of which collectively shape the delicate balance between infection and immunity.

In terms of active defense, the host initiates clearance programs against different pathogens through specific p62 modification events. In antiviral defense, against DNA virus infection, the host can mediate phosphorylation of p62 at S403 via the TRIM23–TBK1 axis, thereby promoting the degradation of viral components via the selective autophagy pathway [79]. To precisely control the intensity of the immune response and prevent excessive and sustained interferon signaling, the host employs multiple feedback mechanisms. For example, after viral infection, OTU domain‐containing protein 7B (OTUD7B) can be induced to perform K63‐linked deubiquitination of p62 at K7, a modification that promotes p62 oligomerization and accelerates the autophagic degradation of the transcription factor IRF3 [127]. Similarly, the virus‐induced E3 ligase TRIM2α can catalyze the ubiquitination of p62 at K91, also promoting the clearance of IRF3 [82]. In antibacterial xenophagy, specific ubiquitination modifications of p62 are crucial. For instance, the E3 ligase RING finger protein 166 (RNF166) mediates K29/K33‐linked polyubiquitination of p62 at Lysine 91, 189, and 313, with K91 ubiquitination thought to potentially enhance p62 function by promoting its oligomerization [148]. Furthermore, the host has evolved more complex active regulatory strategies. In specific viral infections, the E3 ligase TRIM25 can function through a dual mechanism: on one hand, it degrades Keap1 to activate the Nrf2 pathway and upregulate p62 expression; on the other hand, it catalyzes K63‐linked ubiquitination of p62 to inhibit its oligomerization and autophagic function, thereby achieving a fine balance against viral replication [149]. This p62‐based defense mechanism is evolutionarily conserved. For example, in lower vertebrates, the TRAF6–p62 axis similarly mediates antibacterial xenophagy [150].

Faced with host defenses, pathogens have evolved precise hijacking strategies to reprogram p62 function by interfering with its PTMs state to achieve evasion. Viruses often rewrite p62's phosphorylation code by hijacking host kinases or expressing their own kinases. For example, the hepatitis B virus surface antigen can bind to and alter the substrate preference of TBK1. While suppressing interferon production, it utilizes this kinase to catalyze phosphorylation of p62 at S403, creating an autophagy‐favorable environment for replication [128]. It can also block autophagosome–lysosome fusion by inhibiting the expression of the key fusion protein synaptosomal‐associated protein 29 (SNAP29), leading to autophagic flux arrest and providing favorable conditions for viral replication [128]. Similarly, members of the Parvoviridae family, such as mink enteritis virus, can promote their own replication by upregulating the expression of the host E3 ligase TRIM23 to activate TBK1, thereby driving p62 S403 phosphorylation [151]. The coronavirus M protein activates the host 3‐phosphoinositide‐dependent protein kinase 1 (PDPK1) to catalyze phosphorylation of p62 at T138, thereby inhibiting antiviral xenophagy and instead aberrantly activating mitophagy to clear immune signaling platforms (e.g., mitochondria) [129]. Similarly, porcine reproductive and respiratory syndrome virus (PRRSV) deploys a dual autophagy strategy to degrade melanoma differentiation‐associated protein 5 (MDA5), a key viral RNA sensor. It upregulates host CK2α to phosphorylate p62 and TRIM21 to ubiquitinate MDA5 at K240, directing MDA5 for p62‐mediated selective autophagy. Concurrently, viral nsp3 enhances MDA5 binding to the ubiquitin‐independent receptor chaperonin containing TCP1 subunit 2 (CCT2), enabling aggrephagy. This dual‐receptor mechanism exemplifies a sophisticated viral strategy of synergistically co‐opting host degradation pathways to achieve profound immunosuppression [152]. Furthermore, driving abnormal nucleocytoplasmic shuttling of p62 is another common evasion mechanism. For instance, vaccinia virus catalyzes phosphorylation of p62 at T269/S272 via its own encoded kinase, driving its nuclear translocation to evade cytosolic autophagic clearance [153]. The host has also evolved corresponding defense mechanisms against such strategies. For example, the neurodevelopmental kinase cyclin‐dependent kinase‐like 5 (CDKL5) can promote p62 aggregation and efficient recognition of the capsid proteins of neurotropic viruses by phosphorylating p62 at T269/S272, subsequently clearing them via the autophagy pathway [154]. In human cytomegalovirus infection, phosphorylation of p62 at S272 promotes its nuclear retention, thereby enhancing its interaction with viral nuclear proteins. This may promote the degradation of viral components, ultimately restricting viral proliferation [155]. Bacteria employ strategies such as directly interfering with host modifying enzymes or catalyzing noncanonical modifications. For example, the actin‐assembly‐inducing protein (ActA) of *Listeria monocytogenes* evades xenophagy by blocking ULK1‐mediated phosphorylation of murine p62 at Serine 409 (corresponding to human S407) [130]. *Salmonella* can inhibit the AKT–mTOR signal via NOD‐like receptor family pyrin domain containing 6 (NLRP6), reducing the phosphorylation level of murine p62 at S351 (corresponding to human S349), thereby weakening the host's Nrf2 antioxidant defense pathway [131]. The *Legionella* effector protein SidE can catalyze noncanonical phosphoribosyl‐linked ubiquitination of p62 at Serine 361 and Serine 365 (S361 and S365), transiently inhibiting the host Nrf2 defense pathway [156]. Parasites can also intervene in this network. For example, the E3 ligase *Trichinella spiralis* RING finger protein (Ts‐RNF) secreted by *Trichinella spiralis* specifically catalyzes K63‐linked polyubiquitination of host p62 at Lysine 422 (K422) within its UBA domain. This modification weakens p62‐mediated mitophagy, thereby interfering with host myocyte differentiation and favoring parasite survival [132]. Even the host's own defense mechanisms may be depleted during persistent infection. For instance, in herpes simplex virus type 1 (HSV‐1) infection, host restriction of viral replication via xenophagy leads to the degradation of autophagy receptors including p62. The viral protein unique short protein 11 (US11) counteracts this by slowing down the ubiquitination and degradation of p62 to evade clearance, reflecting a tug‐of‐war between both sides at the level of PTMs dynamics [157].

### 3.5 Regulatory Role of the p62 PTMs Network in Inflammatory and Autoimmune Diseases

In the pathological progression of various inflammatory and autoimmune diseases, dysregulation of the p62 PTMs network is a key molecular event driving immune imbalance and tissue damage. The abnormal modification state of p62 directly leads to excessive or sustained immune activation by disrupting precise negative feedback regulation.

In the development of systemic autoimmune diseases, aberrant modifications of p62 can lead to the pathological persistent activation of the Type I interferon pathway. For instance, in systemic lupus erythematosus (SLE), the E3 ligase TRIM21 catalyzes K63‐linked polyubiquitination of p62. This modification blocks the normal binding of p62 to STING, causing abnormal intracellular accumulation of the STING signaling molecule, thereby driving the persistent activation of the Type I interferon pathway and exacerbating autoimmune pathology [133]. In contrast, the host's negative feedback mechanisms for suppressing excessive immunity may be ineffective or insufficient under pathological conditions. Although there exists a physiological feedback pathway involving CK1‐mediated phosphorylation of murine p62 at S351 (corresponding to human S349) to promote STING clearance [81], this feedback may be inadequate to counteract sustained abnormal activation in disease environments such as SLE.

In models of acute inflammatory injury, imbalance in p62 acetylation directly affects the intensity and outcome of the inflammatory response. Histone deacetylase 10 (HDAC10)‐mediated deacetylation of p62 at Lysine 165 (K165) in macrophages excessively stabilizes the p62 protein and aberrantly activates autophagic flux. This process not only fails to alleviate inflammation but may instead exacerbate tissue damage [134]. Conversely, in protective mechanisms that suppress excessive inflammation, deacetylation of p62 by the deacetylase Sirt1 can promote the clearance of ROS. Weakening of this function may be associated with uncontrolled inflammation [83]. Furthermore, the Cullin 3 complex, which negatively regulates inflammatory signaling, promotes p62 degradation via K48‐linked ubiquitination. Impairment of this pathway may lead to sustained activation of inflammatory signaling pathways such as interleukin‐1 beta (IL‐1β) [158].

In tissue‐specific chronic inflammatory and degenerative diseases, dysregulation of the p62 PTMs network contributes to disease progression. In the pathological environment of osteoarthritis, the inflammatory cytokine IL‐1β can upregulate p62 expression via the mitogen‐ and stress‐activated protein kinase 1 (MSK1)–NF‐κB p65 pathway. The protective mitophagy promoted by p62 serves as a compensatory mechanism for chondrocytes under stress, and failure of this mechanism may accelerate cartilage degeneration [135]. In the context of neuroinflammation and spinal cord injury, aconitate decarboxylase 1 (ACOD1)‐mediated phosphorylation of murine p62 at S351 in microglia and its subsequent activation of the Keap1–Nrf2 antioxidant pathway constitute a crucial endogenous protective program for limiting neuroinflammation. Impairment of this function may worsen neural damage [159].

It is noteworthy that the failure of the precise feedback mechanisms that control the duration of immune responses can itself form the pathological basis. For example, following cGAS–STING pathway activation, TBK1‐mediated phosphorylation of p62 at S403 triggers the autophagic degradation of STING, representing a classic negative feedback loop [80]. Meanwhile, the ubiquitin‐like protein HLA‑F adjacent transcript 10 (FAT10), produced during early immune responses, targets and promotes p62 degradation via the proteasomal pathway through mono‐FAT10ylation modification catalyzed by the E1/E2 enzyme system involving ubiquitin‐like modifier activating enzyme 6 (UBA6) and UBA6‐specific E2 enzyme 1 (USE1). This constitutes another dynamic feedback model to control the duration of the interferon response [32]. Reduced efficiency or loss of specificity in these feedback mechanisms serves as an important molecular basis for the persistence of chronic inflammation and autoimmunity.

### 3.6 Dysregulation and Intervention of the p62 PTMs Network in Muscle‐Related Diseases

In acquired muscle dysfunction, the functional state of the p62 PTMs network directly determines the direction of disease progression and the fate of muscle tissue. Its pathological significance is often reflected in the initiation and ultimate failure of protective compensatory responses.

In cancer cachexia, progressive skeletal muscle atrophy is closely associated with p62 PTMs‐mediated systemic antioxidant stress. Under pathological conditions, endogenously produced IL‐1β in skeletal muscle is upregulated, which subsequently activates the transcription factor Nrf2 via the “IL‐1β–NBR1–p62 S351 phosphorylation” axis, driving the expression of downstream antioxidant enzymes. This constitutes an early compensatory protective response that resists catabolism and counteracts mouse muscle atrophy [136, 137]. However, during the sustained progression of cachexia, this protective pathway often shows insufficient compensation or activation failure. When the Nrf2 signaling mediated by p62 phosphorylation cannot be adequately initiated or maintained, the muscle loses its crucial antioxidant defense, thereby accelerating the atrophy process.

Similarly, in skeletal muscle dysfunction associated with chronic obstructive pulmonary disease, the p62‐mediated Keap1–Nrf2 antioxidant pathway also faces functional impairment or insufficient compensatory activation. Systemic oxidative stress persists, while endogenous activation events such as p62 phosphorylation are inadequate to sustain effective antioxidant defense, leading to the accumulation of oxidative damage in muscle and functional decline. Research indicates that one of the mechanisms underlying the improvement in muscle function brought about by exercise intervention is the further promotion of p62 phosphorylation, thereby amplifying this compensatory pathway and enhancing the endogenous antioxidant capacity of skeletal muscle [69]. This conversely confirms that the insufficient activation level of p62 PTMs under baseline pathological conditions is an important mode of its involvement in disease pathology.

Therefore, in acquired muscle diseases, the pathological role of p62 PTMs presents a dynamically continuous evolution: from an initial protective compensatory signal to a state of relative or absolute functional insufficiency under sustained stress. The dose and timing of its activation directly influence muscle survival and function.

### 3.7 The Protective Mechanism of p62 in Ischemic Stroke/Cerebral Ischemia–Reperfusion Injury

In acute ischemic brain injury (stroke/cerebral ischemia–reperfusion), the proper activation of the p62 PTMs network is a prerequisite for its neuroprotective function. During the pathological process, insufficient induction, dysregulation, or functional failure of p62 PTMs directly exacerbates neuronal damage.

On one hand, phosphorylation of p62 at S403 is necessary for initiating effective mitophagy. Research reveals that timely activation of the Parkin–TBK1–p62 signaling axis after cerebral ischemic injury can promote p62 S403 phosphorylation, thereby enhancing its recognition and clearance of ubiquitinated mitochondria. This is crucial for restoring mitochondrial quality control and mitigating hippocampal neuronal damage [138]. However, under severe ischemic stress, this axis may fail to be adequately activated due to impaired upstream signaling, preventing p62 from executing its critical scavenger function. Consequently, damaged mitochondria accumulate, exacerbating oxidative stress and cell death.

On the other hand, the protein stability of p62 is precisely regulated by ubiquitination, and its stability forms the foundation for activating broad cellular defense programs. In hypoxic–ischemic brain injury, exosome‐derived miR‐653‐3p can block the ubiquitin‐mediated degradation of p62 by inhibiting the E3 ubiquitin ligase TRIM21, thereby stabilizing p62 protein. This stabilization subsequently activates the Nrf2 antioxidant pathway and downstream anti‐inflammatory programs [139]. Under pathological conditions, if this stability regulation fails (e.g., due to abnormally active TRIM21 or insufficient delivery of protective miRNA), p62 will be excessively degraded. This renders the cell unable to initiate adaptive responses via this pathway, making it more vulnerable to oxidative and inflammatory attacks.

### 3.8 Decompensation of the p62 PTMs Network in Chronic Stress and Cardiac Aging

In the development of age‐related heart disease, the function of p62 exhibits a dynamic evolution from adaptive protection to decompensatory collapse, with the state of its PTMs network being the central hallmark of this transition. A study based on a transgenic mouse model with cardiac‐specific overexpression of adenylyl cyclase 8 systematically reveals this process [140].

During the early stage of chronic cyclic adenosine monophosphate (cAMP) stress (in young mice), the heart achieves successful compensation by activating the p62 network: autophagic flux is enhanced, and the levels of p62 protein and its phosphorylation at S351 and Serine 405 (S405, corresponding to S403 of human p62) are upregulated. Among these, S351 phosphorylation aids in activating the Keap1–Nrf2 antioxidant pathway, while S405 phosphorylation enhances p62's clearance capacity for ubiquitinated cargo (such as damaged mitochondria). Together with the concurrently elevated proteasome activity, they cooperatively maintain a high functional state and proteostasis in the heart.

However, under the dual impact of aging and prolonged stress (in aged mice), this precise network ultimately fails. At this stage, the heart exhibits comprehensive proteostasis collapse: impaired autophagic flux, decreased proteasome activity, and fragmentation of the mitochondrial network. Correspondingly, p62 and LC3 form abnormal insoluble inclusions that accumulate massively, accompanied by the deposition of lipofuscin and desmin preamyloid oligomers. At this point, aberrant p62 phosphorylation has become both a direct manifestation of autophagic flux obstruction and a driving factor in a vicious cycle. On one hand, phosphorylated p62–ubiquitinated protein complexes are abnormally retained due to the blocked autophagy–lysosomal degradation pathway, forming insoluble inclusions. On the other hand, these persistently existing abnormal aggregates inefficiently deplete the autophagy machinery and serve as scaffolds for the deposition of lipofuscin and misfolded proteins (such as desmin oligomers). Together, they create a proteotoxic environment that is difficult to clear, directly impairing cardiomyocyte structure and function. This transition from “efficient adaptation” to “decompensatory collapse” directly leads to dilated cardiomyopathy and cardiac failure.

### 3.9 Functional Impairment of the p62 PTMs Network in Renal Fibrosis

In the development of renal fibrosis, dysregulation of specific ubiquitination modifications of p62 is a key molecular event driving the pathological process. Studies have shown that the expression of the E3 ubiquitin ligase TRIM21 is significantly upregulated in fibrotic tissue [141]. TRIM21 directly catalyzes the K63‐linked polyubiquitination of p62. Unlike the K48‐linked modifications that mediate degradation, this K63 ubiquitination does not affect the protein stability of p62. Instead, it directly weakens the core function of p62 as an autophagy receptor—the ability to effectively aggregate and deliver substrates to the autophagic degradation pathway—by inhibiting its oligomerization. The pathological consequence of this modification is clear: functionally impaired p62 cannot mediate the effective autophagic clearance of the core profibrotic factor transforming growth factor‑beta 1 (TGF‐β1), leading to abnormal intracellular accumulation of TGF‐β1. The persistently activated TGF‐β1 signaling then drives the overactivation of the downstream mothers against decapentaplegic homolog (SMAD) pathway, ultimately contributing to the pathological deposition of extracellular matrix and the progression of fibrotic lesions. Genetic evidence further confirms that deletion of TRIM21 can restore normal oligomerization and function of p62, reversing the aforementioned molecular phenotypes. Therefore, the dysregulation of the TRIM21–p62 K63 ubiquitination–TGF‐β1 axis is a central link connecting abnormal p62 PTMs with the pathogenesis of renal fibrosis. It elucidates the molecular pathway through which the functional state of p62 is reprogrammed via specific PTMs, thereby directly participating in the establishment of fibrotic disease.

## 4 From Mechanisms to Translation: Preclinical Models and Therapeutic Potential

The PTMs status of p62 is not only a key regulatory node in disease progression but also a potential therapeutic intervention target. Preclinical studies based on animal models provide proof‐of‐concept and direct evidence for developing pharmacological strategies to precisely modulate specific p62 PTMs for disease treatment (Table 2).

**TABLE 2:** Summary of preclinical intervention strategies targeting p62 PTMs.

Disease category	Intervention/agent	Targeted PTMs/mechanism	Preclinical model	Key outcome	References
Oncology	JMA1 (stapled peptide)	Inhibits EGFR‐driven p62 Y433 phosphorylation	Lung cancer xenograft model	Restored autophagy function; significantly inhibited tumor growth	[55]
	Chrysanthemum indicum extract	Downregulates p62 protein levels and S349 phosphorylation	Cutaneous squamous cell carcinoma mouse model (UV‐induced)	Reversed carcinogenic sustained Nrf2 activation; prevented tumor progression	[160]
	K67 (small molecule)	Disrupts pS349‐p62/Keap1 interaction	Hepatocellular carcinoma models (in vitro and in vivo)	Inhibited tumor cell proliferation; reversed chemoresistance	[92, 101, 161]
	Tiliroside (natural compound)	Inhibits TBK1 kinase, suppressing p62 S349 phosphorylation	Hepatocellular carcinoma model	Restored Keap1‐mediated Nrf2 degradation; induced ferroptosis; sensitized tumors to sorafenib	[162]
Metabolic liver disease	Ezetimibe (AMPK activator)	Induces hepatic p62 S349 phosphorylation	Diet‐induced NASH mouse model	Activated Nrf2 pathway; alleviated hepatic steatosis, inflammation, and fibrosis	[67]
	Niclosamide (AMPK activator)	Induces hepatic p62 S349 phosphorylation	Lipotoxicity mouse model	Activated Nrf2 pathway; protected against diet‐induced NASH	[163]
	La(NO_3_)_3_ (lanthanum nitrate)	Promotes p62 phosphorylation, enhancing Keap1 sequestration	Acute alcoholic liver injury mouse model	Activated Nrf2 pathway; exerted hepatoprotective effects	[164]
	MG‐CH (ammonium glycyrrhizinate/cysteine)	Induces p62 S351 phosphorylation, promoting Keap1 autophagic degradation	CCl_4_‐induced liver fibrosis model	Activated Nrf2; exerted antifibrotic effects	[165]
Neurodegenerative diseases	LRRK2 kinase inhibitors (under development)	Corrects p62 phosphorylation network imbalance (restore S349/S403, reduce T138)	Parkinson's disease models	Potential neuroprotective effects (proposed)	[74, 116]
	Memantine/ifenprodil (NMDA receptor antagonists, repurposed)	Upregulates p62 S349 phosphorylation; activates autophagy	Retinal pigment epithelial cells (A2E‐induced toxicity model)	Cleared toxic metabolite A2E; potential therapy for age‐related macular degeneration	[166]
Infectious diseases	SMIP‐031 (small molecule)	Inhibits PPM1A phosphatase, stabilizing/elevating p62 S403 phosphorylation	Mycobacterium tuberculosis‐infected macrophages; mouse model	Restarted antibacterial autophagy; reduced bacterial load in mice	[167]
	Bardoxolone methyl (CDDO‐Me) (Nrf2 activator)	Induces p62 S349 phosphorylation, promoting Keap1 degradation	Rabies virus‐infected cells (in vitro)	Activated Nrf2 pathway; inhibited viral replication (efficacy close to ribavirin)	[168]
	High‐frequency repetitive magnetic stimulation (rMS) (physical intervention)	Increases p62 protein and S349 phosphorylation levels	Staphylococcus aureus‐infected macrophages; mouse model	Activated p62–Keap1–Nrf2 axis; enhanced antibacterial capacity; suppressed inflammation; promoted bacterial clearance	[169]
Autoimmune diseases	SSTC3 (CK1 agonist)	Promotes p62 S351 phosphorylation, accelerating STING autophagic degradation	Cellular models of cGAS–STING activation	Suppressed aberrant interferon responses; potential strategy for autoimmune diseases	[81]
Muscle degenerative diseases	Ectoine (osmoprotectant)	Upregulates p62 S366 phosphorylation; improves autophagic flux	Duchenne muscular dystrophy (mdx) mouse model	Improved autophagic flux indicators in skeletal muscle	[170]
Cardiovascular Injury	Bitter melon‐derived nanovesicles	Inhibits K48 ubiquitination‐mediated degradation of p62, stabilizing the protein	Doxorubicin‐induced cardiotoxicity model	Stabilized p62; activated Nrf2 pathway; exerted cardioprotective effects	[171]
Kidney diseases	Astragaloside IV	Upregulates p62 phosphorylation, activating Nrf2‐mediated antioxidant response	Chronic kidney disease model (1‐deoxysphinganine‐induced)	Alleviated mitochondrial dysfunction and oxidative damage in renal tubular cells	[172]
	Carnosol (natural compound)	Binds to and activates mTOR, inducing p62 S349 phosphorylation, promoting Keap1 autophagic degradation	Hypertensive renal injury model	Activated Nrf2; alleviated renal oxidative stress and injury	[173]
Aging/reproductive senescence	Bacteroides fragilis‐derived extracellular vesicles (miR‐1246)	Inhibits SKP2 E3 ligase, reducing K48‐linked polyubiquitination of p62, stabilizing the protein	Ovarian aging mouse model	Stabilized p62; activated Nrf2; improved ovarian function; delayed aging	[174]
Skin barrier function	PEG‐23 glyceryl distearate (GDS‐23) (nonionic surfactant)	Induces p62 S349 phosphorylation, activating Nrf2 pathway	Epidermal keratinocytes	Promoted expression of barrier proteins (filaggrin, loricrin), moisturizing factors (AQP3), and antioxidant proteins; enhanced skin antioxidant defense	[175, 176]

*Abbreviations*: JMA1, a stapled peptide inhibitor; A2E, N‐retinylidene‐N‐retinylethanolamine; AMPK, AMP‐activated protein kinase; CCl4, carbon tetrachloride; CDDO‐Me, bardoxolone methyl; CK1, casein kinase 1; DMD, Duchenne muscular dystrophy; EGFR, epidermal growth factor receptor; GDS‐23, PEG‐23 glyceryl distearate; K67, a small‐molecule inhibitor; Keap1, Kelch‐like ECH‐associated protein 1; LRRK2, leucine‐rich repeat kinase 2; MG‐CH, ammonium glycyrrhizinate/cysteine compound; mTOR, mechanistic target of rapamycin; NASH, nonalcoholic steatohepatitis; NMDA, N‐methyl‐D‐aspartate; Nrf2, nuclear factor erythroid 2‐related factor 2; PEG‐23, polyethylene glycol (23 units); PPM1A, protein phosphatase 1A; rMS, repetitive magnetic stimulation; S349, Serine 349; S351, Serine 351; S366, Serine 366; S403, Serine 403; SKP2, S‐phase kinase‐associated protein 2; STING, stimulator of interferon genes; T138, Threonine 138; TBK1, TANK‐binding kinase 1; Y433, Tyrosine 433.

In the field of oncology, a specific fusion peptide inhibitor, JMA1, was designed to target p62 Y433 phosphorylation driven by aberrant EGFR activation in lung cancer. This inhibitor successfully blocked p62 Y433 phosphorylation, restored autophagy function, and significantly inhibited tumor growth in xenograft models, achieving precise intervention against a specific oncogenic PTM of p62 [55]. In a mouse model of cutaneous squamous cell carcinoma, *Chrysanthemum indicum* extract demonstrated preventive potential by downregulating p62 protein levels and its S349 phosphorylation, thereby reversing carcinogenic sustained Nrf2 activation [160]. The natural compound tiliroside was identified as a potent TBK1 inhibitor that suppresses p62 S349 phosphorylation, thereby restoring Keap1‐mediated Nrf2 degradation, inducing ferroptosis, and sensitizing hepatocellular carcinoma cells to sorafenib in preclinical models [162]. Beyond strategies targeting specific phosphorylation sites, broadly targeting the p62–Keap1 interaction is another important approach. For instance, the small‐molecule compound K67, as a noncovalent binding inhibitor, effectively blocked the binding of p62 to Keap1, thus inhibiting the persistent Nrf2 activation driven by p62 S349 phosphorylation. In preclinical liver cancer models, K67 not only inhibited tumor cell proliferation but also reversed its resistance to chemotherapeutic drugs, further demonstrating the therapeutic value of targeting this PTM axis [92, 101, 161].

In metabolic liver disease, treatment of nonalcoholic steatohepatitis models with AMPK activators, ezetimibe or niclosamide, induced hepatic p62 S349 phosphorylation, subsequently activating the Nrf2 antioxidant pathway and effectively alleviating hepatic steatosis, inflammation, and fibrosis in mouse models [67, 163]. Additionally, in an acute alcoholic liver injury model, the rare earth element lanthanum nitrate (La(NO_3_)_3_) exerted hepatoprotective effects by specifically promoting p62 phosphorylation, enhancing its sequestration of Keap1 and activating the Nrf2 pathway [164]. For neurodegenerative diseases, LRRK2 kinase inhibitors may exert neuroprotective effects in PD models by correcting imbalances in the p62 phosphorylation network (e.g., restoring S349/S403 phosphorylation while reducing abnormally elevated T138 phosphorylation), although specific drugs are still under development [74, 116]. In ophthalmic degenerative diseases, clinically used N‐methyl‐D‐aspartate (NMDA) receptor antagonists, memantine and ifenprodil, were found to significantly upregulate p62 S349 phosphorylation levels and activate autophagy in retinal pigment epithelial cells, effectively clearing the toxic metabolite N‐retinylidene‐N‐retinylethanolamine (A2E) closely associated with age‐related macular degeneration pathogenesis [166]. This provides direct evidence for drug repurposing by pharmacologically activating p62‐mediated autophagy to treat retinal degenerative diseases.

In infectious diseases, progress has been made in host‐directed therapy for *Mycobacterium tuberculosis*. The small‐molecule compound SMIP‐031, by inhibiting the dephosphatase protein phosphatase 1A (PPM1A), stabilized and elevated p62 S403 phosphorylation levels in macrophages, thereby restarting antibacterial autophagy and effectively reducing bacterial load in mice [167]. Furthermore, direct evidence is provided for antiviral treatment: the potent Nrf2 activator, bardoxolone methyl, significantly inhibited rabies virus replication in vitro by inducing p62 S349 phosphorylation and promoting Keap1 degradation to activate the Nrf2 pathway. Its efficacy was close to that of the traditional antiviral drug ribavirin, offering a new strategy for pharmacologically activating the p62–Keap1–Nrf2 axis against viral infection [168]. Beyond small‐molecule drugs, physical interventions also show potential for modulating p62 to treat infections. Research found that high‐frequency repetitive magnetic stimulation (rMS) could significantly increase p62 protein and its S349 phosphorylation levels in macrophages without causing oxidative stress. This subsequently enhanced cellular antioxidant and direct antibacterial capabilities by activating the p62–Keap1–Nrf2 axis, effectively suppressing inflammation and promoting bacterial clearance in a *Staphylococcus aureus* infection model [169].

The therapeutic potential of p62 phosphorylation regulation has also extended to the field of autoimmune diseases. Strategies targeting its upstream regulatory kinases show unique value. For example, the CK1 agonist, SSTC3, confirmed to mediate the degradation of the key innate immune signaling adaptor STING by promoting murine p62 S351 phosphorylation, has been proposed as a potential strategy to alleviate autoimmune‐related abnormal interferon responses [81].

For muscular degenerative diseases, the osmoprotectant Ectoine was confirmed to upregulate p62 S366 phosphorylation levels in skeletal muscle of the Duchenne muscular dystrophy (DMD) *mdx* mouse model and simultaneously improve autophagic flux indicators [170].

Furthermore, in cardiovascular injury and fibrotic diseases, bitter melon‐derived nanovesicles stabilized p62 protein by inhibiting its K48 ubiquitination‐mediated degradation, subsequently activating the Nrf2 pathway to exert cardioprotective effects [171]. In a liver fibrosis model, the compound drug ammonium glycyrrhizinate/cysteine (MG‐CH) exerted antifibrotic effects by inducing p62 S351 phosphorylation and promoting the autophagic degradation of Keap1, thereby activating Nrf2 [165]. Specifically, in liver fibrosis models, the compound drug MG‐CH was confirmed to act via a noncanonical mechanism: it specifically induced phosphorylation of p62 at S351, enhanced p62 binding to Keap1, and simultaneously activated autophagic flux, directing Keap1 sequestered by p62 into autolysosomes for degradation. This mechanism of “phosphorylated p62‐mediated autophagic degradation of Keap1” effectively activated the Nrf2 antioxidant pathway, thereby exerting antifibrotic effects [165].

In a chronic kidney disease model, astragaloside IV was confirmed to alleviate mitochondrial dysfunction and oxidative damage caused by the accumulation of the toxic metabolite 1‐deoxysphinganine through upregulating p62 phosphorylation levels in renal tubular cells, activating the Nrf2‐mediated antioxidant response [172]. Similarly, in a hypertensive renal injury model, the natural compound carnosol was confirmed to directly bind to and activate mTOR, subsequently specifically inducing phosphorylation of p62 at S349. This promoted the autophagic degradation of Keap1, activated the Nrf2 antioxidant pathway, and ultimately alleviated renal oxidative stress and injury [173].

Latest research further indicates that the p62 PTMs network is a key target for regulating organ‐specific aging. In an ovarian aging model, the gut probiotic *Bacteroides fragilis* delivered miR‐1246 to ovarian cells via its derived extracellular vesicles, targeting and inhibiting the expression of the E3 ubiquitin ligase S‐phase kinase‐associated protein 2 (SKP2). This directly reduced K48‐linked polyubiquitination of p62 protein, thereby blocking its degradation via the proteasomal pathway and stabilizing p62 protein levels. The accumulated p62 protein competitively bound to Keap1, subsequently activating the Nrf2‐mediated antioxidant transcriptional program, ultimately improving ovarian function and delaying aging [174]. This discovery establishes a gut–ovary axis signaling pathway, providing a novel therapeutic strategy for intervening in age‐related reproductive decline by regulating p62 stability through oral probiotics or targeted miRNA delivery. Furthermore, the regulation of p62 phosphorylation also shows clear therapeutic potential in skin barrier function repair. Studies confirmed that the nonionic surfactant PEG‐23 glyceryl distearate (GDS‐23) could induce p62 S349 phosphorylation, subsequently activating the Nrf2 pathway. This significantly promoted the expression of key barrier proteins (e.g., filaggrin, loricrin), moisturizing factors (e.g., aquaporin 3), and antioxidant proteins in epidermal keratinocytes, thereby enhancing skin antioxidant defense [175, 176]

## 5 Conclusion and Prospects for the p62 PTMs Network

### 5.1 p62: A Molecular Integrator Programmed by PTMs Codes

As a critical intracellular signaling hub protein, the precise regulation of p62 function largely depends on a complex PTMs network (Figures 3 and S1). A salient feature is that its multiple key phosphorylation sites are generally regulated by different upstream signaling pathways and kinases. This many‐to‐one design pattern is the core mechanism enabling its signal integration and context decoding (Figure 4). The S349 site located in the KIR domain is the most typical example. It serves as a common substrate for over seven kinases (including mTOR, AMPK, ULK1, TAK1, CK1, PKC‐δ, and PLK2), integrating multidimensional signals such as nutrient status, energy levels, autophagic activity, and oxidative stress like a molecular processor, thereby initiating programmed cellular defense responses via activation of the Keap1–Nrf2 axis. At the same time, the same S349 phosphorylation event can be driven by completely different kinases in different tumor cells or microenvironmental regions, leading to highly heterogeneous functional outputs. For example, in nutrient‐rich tumor core regions, mTORC1 predominantly phosphorylates S349 to couple anabolic metabolism with antioxidant defense [4, 11, 76, 77]. In energy‐starved tumor edge regions, AMPK takes over this modification to initiate metabolic adaptation [67]. In oxidative stress regions, the DNA damage response kinase CHK2 phosphorylates S349 to coordinate genome protection with antioxidant defense [68]. In inflammation‐rich microenvironments, TAK1 mediates S349 phosphorylation to integrate proinflammatory signals [66]. More importantly, kinase‐specific S349 phosphorylation does not occur in isolation but tends to form specific combinations with different co‐modification networks, thereby producing distinct biological outcomes. Under energy stress, AMPK‐mediated S349 phosphorylation often co‐occurs with S403 phosphorylation; the former activates antioxidant defense, while the latter initiates mitophagy [11, 28, 43], together constituting a coupled module of metabolic adaptation and mitochondrial quality control that helps cells survive energy crises. In contrast, under nutrient‐rich conditions, mTORC1‐mediated S349 phosphorylation forms a positive feedback loop with T269/S272 phosphorylation, locking growth signals and antioxidant defense into a self‐reinforcing proanabolic circuit [11, 76]. Furthermore, under sustained proteotoxic stress, PLK2‐mediated S349 phosphorylation tends to cooperate with T269/S272 phosphorylation to drive the transition of p62 aggregates from a dynamic to a solid‐like state, promoting sequestration rather than degradation of toxic proteins [59, 76]. This kinase‐specific co‐modification network difference reveals the core mechanism by which p62 functions as a signal integration platform: different kinases act as molecular keys that open the same S349 lock, but the functional instruction output depends on the cellular context in which this key operates and the other locks it simultaneously turns, meaning the co‐modification sites.

**FIGURE 3:**
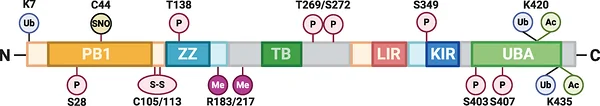
Central PTMs hub of p62/SQSTM1. Schematic representation of the major posttranslational modification sites distributed across the domain architecture of p62/SQSTM1. Key phosphorylation, ubiquitination, acetylation, methylation, S‐nitrosylation, and disulfide‐linked cysteine modification sites are mapped across the overall domain architecture of p62/SQSTM1.

**FIGURE 4:**
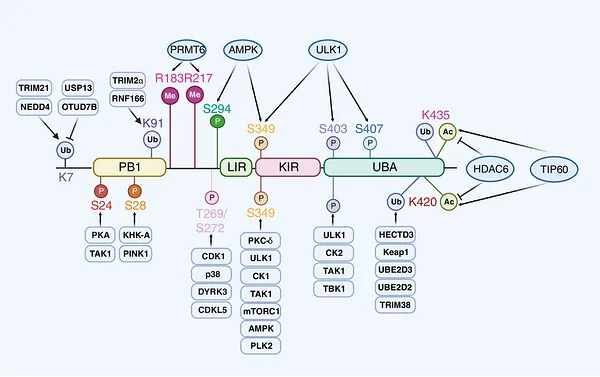
Enzyme‐substrate regulatory map of p62 PTMs. The PTMs regulatory network of p62/SQSTM1 exhibits “many‐to‐one/one‐to‐many” features: multiple modifying/de‐modifying enzymes can act on the same site (many‐to‐one), and a single enzyme can target multiple sites (one‐to‐many). In addition, a single site can undergo multiple types of modifications, together forming a complex regulatory network.

This principle is corroborated at multiple sites. The S403 site in the UBA domain, a key switch regulating autophagic substrate recognition, can be phosphorylated by at least three important kinases: CK2 primarily responds to proteasome inhibition‐induced proteotoxic stress [45]; TBK1 plays a central role in immune activation, such as the cGAS–STING pathway, and mitophagy [28, 80]; and ULK1 is recruited under energy stress through AMPK activation [42, 177]. Similarly, the S28 site in the PB1 domain is also under dual regulation: PINK1 phosphorylates it under proteotoxic stress to redirect ubiquitinated substrates to the autophagy pathway [113], whereas under oxidative stress, KHK‐A phosphorylates S28 to promote p62 oligomerization and Nrf2 pathway activation, supporting cell survival [73]. Furthermore, the T269/S272 site also converges cell cycle and nutrient signals: Cdk1 modifies it during mitosis to regulate cell cycle progression [93], while the p38 kinase family, for example, p38δ, phosphorylates this site upon amino acid signaling, thereby activating the downstream mTORC1 signaling pathway [76].

It is noteworthy that this precise “many‐to‐one” regulatory logic is not confined to phosphorylation but also permeates other p62 modification networks such as ubiquitination and acetylation. At the ubiquitination level, the same K7 site can be targeted by different E3 ligases, yet its functional output is highly dependent on upstream signals and cellular context. Under oxidative stress, TRIM21 catalyzes K63‐linked polyubiquitination at the K7 site, which negatively regulates the Keap1–Nrf2 pathway by inhibiting p62 oligomerization, acting as a molecular brake for the antioxidant response [30]; whereas under specific metabolic or growth signals, neural precursor cell expressed developmentally downregulated protein 4 (NEDD4) mediates ubiquitination at the same K7 site, promoting p62's autophagic function [178]. Similarly, ubiquitination at the K420 site can be directly catalyzed by the E2 enzymes UBE2D2/3 or mediated by the E3 ligase Keap1/Cullin 3 complex, both of which enhance p62's substrate recognition capacity [48, 49]. The former may primarily respond to ubiquitin stress, while the latter is more closely associated with redox status. At the acetylation level, K420 and K435 are acetylated by TIP60 to promote phase separation and autophagy, and deacetylated by HDAC6 to reverse this process [2]; whereas the K295 site is subject to functionally opposing regulation by general control nonderepressible 5 (GCN5) and Sirt1 (GCN5‐mediated acetylation enhances p62 protein turnover, while Sirt1‐mediated deacetylation promotes its stability), thereby precisely controlling the homeostatic pool of p62 [98]. This indicates that each major type of PTMs on p62 constructs a fine‐tuned regulatory network composed of “multiple sites and multiple enzyme systems.” More importantly, similar to S349 in phosphorylation, these modifications are not simple on‐off switches, but rather “context‐dependent molecular instructions” written by different enzymes under specific cellular backgrounds. The same modification type on the same residue can produce distinctly different functional outputs depending on the writing enzyme and the differential co‐modification partners. This multilayered, multienzyme regulatory architecture collectively ensures that p62 can precisely respond to and integrate complex cellular signals from multiple dimensions including metabolism, stress, and immunity. Complementing the many‐to‐one mode is the equally crucial one‐kinase‐to‐multiple‐sites regulatory logic. This mode reveals a core biological principle: by activating a single core kinase, the cell can perform multilayered, coordinated functional programming of p62, thereby efficiently and harmoniously executing specific cellular instructions. For example, the autophagy‐initiating kinase ULK1 can not only phosphorylate S349 to couple autophagy induction with Nrf2 antioxidant pathway activation [11, 24] but also directly phosphorylate two key sites in the UBA domain, S403 and S407 [42, 43]. It synergistically amplifies p62's autophagy receptor function by enhancing ubiquitin chain binding (S403) and dissociating UBA dimers (S407), ensuring robust initiation of the autophagy program. Similarly, the energy sensor AMPK, besides activating antioxidant defense by phosphorylating S349 [67], can also modify S294 to promote mitophagy [147], thus coordinating transcriptional adaptation and organelle cleanup during energy crisis. Therefore, one kinase to multiple sites regulation allows core signaling nodes like ULK1 or AMPK to act as molecular conductors, synchronously modulating multiple functional interfaces of p62. This transforms a single upstream instruction (e.g., initiate autophagy or cope with energy crisis) into a coordinated, multidimensional, and potent downstream execution program, ensuring the integrity and efficiency of the cellular response.

This multidimensional, multilayered PTMs regulatory network endows p62 with remarkably context‐sensitive signal‐processing capacity. Its essence lies in the cell's ability to encode different types of upstream signals, including metabolic status, oxidative pressure, immune alerts, and cell cycle stage, onto specific phosphorylation sites of p62 by selectively activating particular kinases. Simultaneously, a single core kinase can achieve multilayered, integrated programming of a unitary instruction by coordinately phosphorylating multiple functional sites. This transforms p62 from a mere passive signal relay into a dynamic signal processor: it can interpret the current environmental context based on the received kinase fingerprint and output the most appropriate biological response—whether to initiate selective autophagy, activate antioxidant programs, or promote cell growth and division. Consequently, the functional output of p62 is not determined by any single type of modification event. Rather, it is precisely programmed by a PTMs code formed through the dynamic combination and crosstalk of various modification types—phosphorylation, ubiquitination, and acetylation—at its different sites. Within this network, distinct modification sites engage in crosstalk to collectively determine p62's functional output in specific cellular contexts (Figure 5). Herein lies the fundamental bottleneck for traditional target‐the‐protein‐itself strategies: p62 is an essential protein for maintaining cellular homeostasis, so directly eliminating or inhibiting it would cause unacceptable toxicity; its pathogenic role often stems from its normal function being hijacked by aberrant signals, not from mutations in itself; and it lacks the traditional small‐molecule‐friendly active pockets, making direct drug development extremely difficult. Therefore, disease intervention targeting p62 must shift from focusing on the p62 protein alone to precisely deciphering and reprogramming its dynamic PTMs code. Targeting the p62 PTMs network is precisely intended to circumvent these obstacles: by intervening in its upstream modifying enzymes or specifically disrupting disease‐causing modification patterns, it may be possible to achieve selective correction of disease‐related functions while maximally preserving its physiological roles.

**FIGURE 5:**
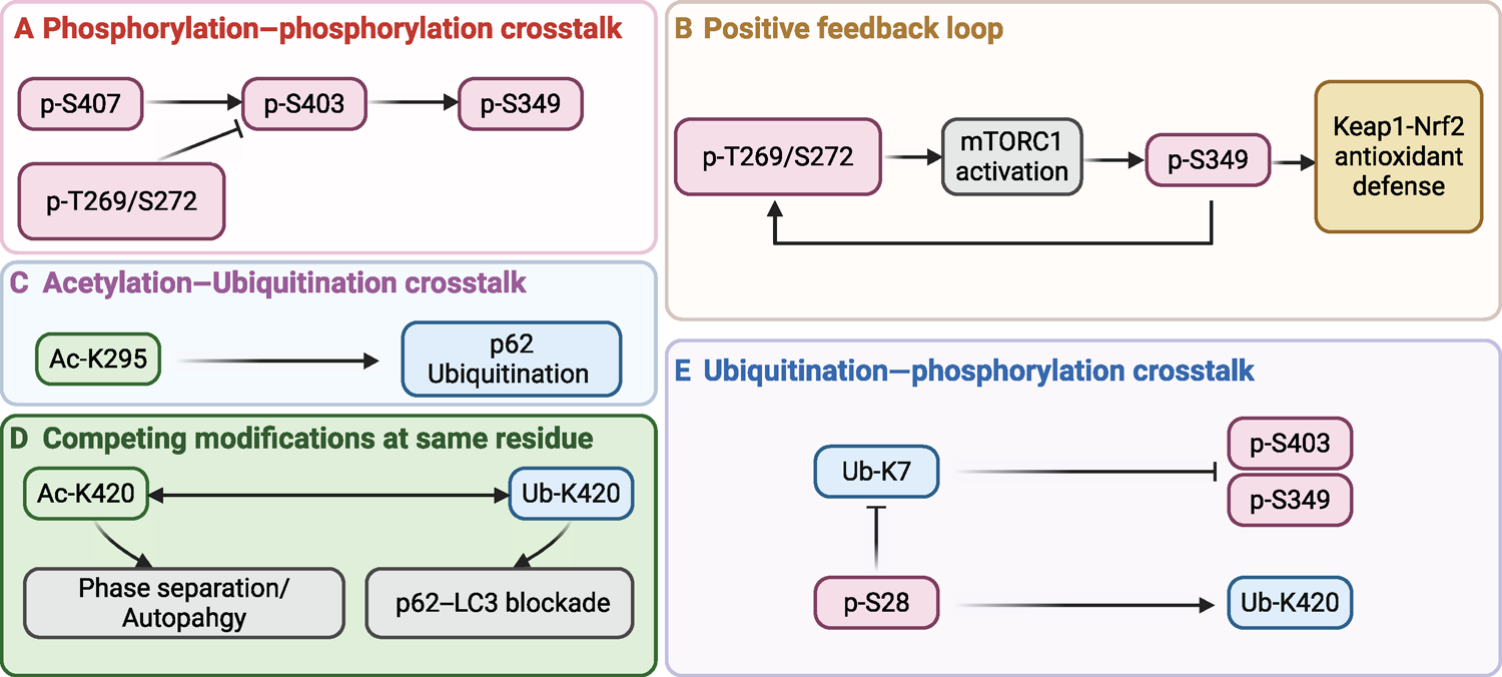
p62 PTMs crosstalk network. The known regulatory relationships among different PTMs on p62 are categorized into five modes of crosstalk. Phosphorylation–phosphorylation crosstalk: (A) Sequential dependence is exemplified by ULK1‑mediated phosphorylation at S407, which relieves UBA auto‑inhibition and permits subsequent S403 phosphorylation. In the context of ALS/FTLD, TBK1 mutation impairs S403 phosphorylation, which in turn compromises S349 phosphorylation. Under sustained proteasome inhibition, phosphorylation at T269/S272 inhibits the prodegradative S403 phosphorylation, shifting p62 function from degradation to sequestration. (B) A positive feedback loop is formed when T269/S272 phosphorylation activates mTORC1, which in turn phosphorylates S349, coupling anabolic signals with the Keap1‑Nrf2 antioxidant defense. (C) Acetylation–ubiquitination crosstalk: Acetylation at K295 (Ac‑K295) suppresses p62 ubiquitination, thereby enhancing p62 protein stability and modulating its downstream signaling output. (D) Competing modifications at the same residue: At K420, TIP60‑mediated acetylation (Ac‑K420) promotes phase separation and autophagy, whereas TRIM38‑mediated K63‑linked ubiquitination (Ub‑K420) creates steric hindrance that physically blocks the p62–LC3 interaction, thereby shutting down autophagic flux. (E) Ubiquitination‑phosphorylation crosstalk: TRIM21‑mediated K63‑linked ubiquitination at K7 (Ub‑K7) negatively regulates multiple phosphorylation events, including S349 and S403. Conversely, KHK‑A‑mediated S28 phosphorylation blocks K7 ubiquitination, forming an antagonistic loop that promotes p62 oligomerization and Nrf2 activation. GSK3β‐mediated S28 phosphorylation triggers TNFAIP1‐mediated ubiquitination at K420, which in turn promotes p62 degradation.

However, successful reprogramming of the p62 PTMs code hinges on understanding its fundamental design principles. Evolution provides a powerful lens for this purpose. Certain modification sites are evolutionarily conserved, underscoring their fundamental and nonreplaceable biological functions. For example, the phase separation‐regulating acetylation sites K420 and K435 are conserved in rodents, indicating that this mechanism has been fixed during mammalian evolution [2]. Conversely, some PTMs exhibit species‐specific divergence, likely reflecting adaptations to particular environmental pressures or physiological complexity. Notably, the oxidation‐sensitive cysteine residues C105 and C113, which mediate disulfide‐dependent oligomerization under oxidative stress, are present in vertebrates but absent in the Drosophila homologue Ref(2)P [50]. Intriguingly, introducing these vertebrate‐specific cysteine residues into Ref(2)P enhances protein turnover and stress resistance in flies, directly demonstrating the functional advantage conferred by this evolutionary acquisition [50]. This coexistence of conservation and divergence provides an important dimension for understanding the p62 PTMs code: conserved sites are essential for maintaining basal homeostatic functions, while species‐specific sites participate in adaptive regulation under particular environmental conditions. This insight has implications for therapeutic strategies: targeting conserved sites may produce broad but potentially riskier effects, whereas targeting species‐specific sites (such as those aberrantly activated only in human diseases) may offer better therapeutic windows. Notably, systematic studies on the evolutionary divergence of p62 PTMs remain scarce, and the cross‐species conservation of most modification sites has yet to be thoroughly investigated. Future research should employ systematic comparative genomics and gain‐of‐function experiments to elucidate the evolutionary trajectories and functional significance of different modification sites. This will help distinguish core regulatory mechanisms from species‐specific adaptations, providing an evolutionary framework for the development of precision therapeutic strategies targeting p62 PTMs.

### 5.2 Constructing a Dynamic Functional Atlas of p62 PTMs: Integrating Technological Pathways and Strategies

The comprehensive analysis of the p62 PTMs network relies heavily on the precise identification of modification sites on p62, and the related technologies have undergone a profound transformation from traditional biochemistry to high‐throughput mass spectrometry. Early approaches depended on chemical or radioactive labeling, which had low throughput and limited resolution, only providing a qualitative answer to “whether p62 is modified.” With the maturation and application of liquid chromatography–tandem mass spectrometry (LC–MS/MS), combined with specific enrichment strategies, researchers began to precisely answer “which residue is modified and what function it serves.” Currently, multimodification sequential enrichment technologies represented by SDS cyclodextrin assisted sample preparation for PTM (SCASP‐PTM) have further broken through the bottleneck of “how multiple modifications cooperate or compete,” moving p62 PTMs research from single‐site identification to dynamic regulatory network mapping. The following sections detail the specific applications and breakthroughs of these technologies in p62 research, organized by developmental stage.

The study of p62 PTMs began in the mid‐1990s. In 1997, Yanagawa et al., using ^32^P‐phosphate metabolic labeling combined with immunoprecipitation, first confirmed that p62 is a physiological phosphoprotein in macrophages [27]. These early works relied entirely on traditional methods such as radioisotope labeling, immunoprecipitation, and in vitro kinase reactions; although they confirmed that p62 could be phosphorylated, they lacked residue‐level precision and could not discover unknown modification types.

The maturation of LC–MS/MS fundamentally changed the landscape of p62 PTMs research. Its core breakthrough lies in the ability to identify specific modification sites directly from digested peptides without prior knowledge of the modification type or kinase identity. The first milestone came in 2011. Matsumoto et al., using LC–MS/MS, identified S403 of p62 as a key phosphorylation site. Combined with site‐directed mutagenesis and phospho‐specific antibodies, they demonstrated that phosphorylation at this site enhances the affinity of the UBA domain for polyubiquitin chains, thereby regulating the selective autophagic clearance of ubiquitinated proteins [45]. In 2016, Pan et al., also using an immunoprecipitation‐coupled LC–MS/MS strategy, enriched Flag‐p62 protein from HEK293T cells, subjected it to sodium dodecyl sulfate–polyacrylamide gel electrophoresis (SDS‐PAGE) separation and in‐gel digestion, and directly performed mass spectrometry analysis, identifying K7 in the PB1 domain of p62 as the TRIM21‐mediated K63‐linked ubiquitination site [30]. LC–MS/MS‐based p62 PTMs research has since flourished. It is noteworthy that the above studies and contemporaneous work mainly relied on immunoprecipitation enrichment of p62 protein followed by direct LC–MS/MS analysis, without additional enrichment of specific modified peptides prior to mass spectrometry, a direct method strategy that remains one of the mainstream approaches for identifying modifications on known proteins.

However, the direct method has limited coverage of low‐abundance modified peptides and is also poor at discovering entirely new modification types. To further expand the depth and breadth of p62 PTMs research, subsequent studies gradually introduced affinity or chemical enrichment strategies targeting different modification types. The core distinction among studies lies in the upstream enrichment approach, that is, choosing the appropriate affinity or chemical enrichment strategy according to the target modification type. For example, acetylation identification mainly employs acetyl‐lysine antibody immunoaffinity enrichment coupled with LC–MS/MS, successfully identifying acetylation sites such as K420 and K435 [2]. Using acyl‐biotinyl exchange enrichment coupled with LC–MS/MS, S‐acylation (palmitoylation) was identified at the C289/C290 dicysteine motif of p62 [35]. Similarly, FAT10ylation was identified by immunoaffinity purification using a novel FAT10 monoclonal antibody followed by LC–MS/MS, further coimmunoprecipitation and colocalization studies validated p62 as a covalent substrate [32]. In contrast, certain modifications, due to their instability, require non‐LC/MS biochemical methods under conventional mass spectrometry conditions. For instance, the identification of the C105–C113 disulfide bond relied entirely on nonreducing SDS‐PAGE and site‐directed mutagenesis. Nonreducing electrophoresis revealed oxidative‐stress‐induced high‐molecular‐weight p62 oligomers that were sensitive to reducing agents. Combined with sequence analysis and structural prediction, C105 and C113 were identified as candidate pairing residues. Finally, the C105S/C113S double mutation abolished disulfide‐dependent oligomerization, confirming that the C105–C113 disulfide bond is the core molecular switch for oxidative‐stress‐induced p62 oligomerization and autophagy activation [50].

Currently, the latest research direction has shifted from simply and crudely detecting multiple modifications to integrated analysis via single‐sample, multimodification sequential enrichment. The SCASP‐PTM technology, which uses cyclodextrin to capture SDS, achieves for the first time the sequential enrichment of multiple modified peptides (e.g., phosphorylated, ubiquitinated) in the same sample without an intermediate desalting step. This approach fundamentally avoids sample inconsistency arising from multibatch parallel experiments and sample loss during desalting steps. Using this technology, Lin et al. revealed for the first time the causal mechanism of p62 degradation induced by poly(I:C): SCASP‐PTM combined with data independent acquisition (DIA) MS enabled quantitative profiling of proteins, phosphorylation sites, and ubiquitination sites in HeLa S3 cells. The data clearly show that p62 degradation depends on GSK3β‐mediated S28 phosphorylation, which in turn triggers TNF alpha induced protein 1 (TNFAIP1)‐mediated ubiquitination at K420, forming a causal cascade from phosphorylation to ubiquitination [25].

The analysis of p62 PTMs is shifting from static, single‐site detection to dynamic, multidimensional, network‐oriented paradigms. Despite these successes, current methodologies for studying p62 PTMs still have significant limitations, underscoring the necessity of technological evolution. First, most methods rely on bulk analysis, averaging out cellular heterogeneity and losing rare events [179]. Second, studies are largely static snapshots, failing to capture the dynamic changes of modifications over time [180]. Third, conventional PTMs identification requires cell lysis, destroying the native spatial context and organelle microenvironment [181]. Fourth, low‐abundance modifications require enrichment strategies that may introduce bias [182]. Finally, establishing causal relationships between specific PTMs and functional outcomes remains challenging, often relying on overexpression systems that may not reflect endogenous dynamics [183]. To address the limitations of traditional methods, recent technologies such as spatial proteomics, proximity labeling, multimodification sequential enrichment, and single‐molecule detection offer systematic solutions; their core principles and potential applications in p62 research are summarized in Table 3. The integrated application of these technologies is expected to propel p62 PTMs research from descriptive modification maps to a new stage of dynamic, network‐based mechanistic dissection.

**TABLE 3:** Comprehensive technological toolbox for dissecting the PTMs regulatory network of p62.

Technique	Core principle	Application in p62 research	Main limitation addressed	References
NO‐IP/MS	Endogenous CRISPR–Cas9 labeling of organellar proteins, mild cell lysis, and antibody capture of intact organelles to preserve native membrane contacts	Isolating autophagosomes, lysosomes, etc., at different functional stages to study p62 modification status and protein composition in each organelle	Traditional subcellular fractionation disrupts native organelle spatial structure and membrane contacts	[184]
CLASP	Chemical crosslinking captures protein pairs within <4 nm to resolve membrane protein topology	Reveals the orientation of specific p62 domains (e.g., UBA, PB1) and their direct interacting partners at the nanoscale	Inability to achieve nanometer‐scale spatial resolution	[185]
TMT	16‐plex isobaric tags label peptides from different conditions; combined single MS run for relative quantification	Comparing dynamic changes of p62 modification levels under different autophagy stages or stress conditions	Batch‐induced quantitative bias	[186]
EAD‐PRM	Electron‐activated dissociation preserves labile modifications; parallel reaction monitoring for targeted validation	Accurate validation of labile modifications on p62 (e.g., acylation)	Loss of unstable modifications under conventional fragmentation	[187]
LC–TIMS–ToF	Ion mobility separates ions by size/shape; orthogonal separation of isomers	Distinguishing coeluting isomeric modified peptides of p62 (e.g., different phosphorylation sites)	Traditional LC–MS cannot resolve isomeric modified peptides	[188]
Conformation‐resolved MS	Ionization of intact proteins under native‐like conditions; preserves higher‐order structure	Directly studying how PTMs (e.g., phosphorylation, ubiquitination) regulate p62 conformation, phase separation, and interaction networks	Disconnection between modification information and protein higher‐order structure/function	[189]
STO‐MS+	DNA used as a large‐mass tag; separation by electrophoresis followed by MS	High‐throughput measurement of PTMs stoichiometry on p62 (e.g., phosphorylation occupancy)	Difficulty in measuring modification stoichiometry	[190]
µPhos	Microplate‐based TiO_2_ enrichment coupled with high‐throughput MS	Parallel quantification of thousands of p62 phosphorylation sites under various conditions	Low throughput and poor reproducibility in phospho‐quantification	[191]
AQUA	Stable isotope‐labeled synthetic peptide internal standard; quantification by MS signal ratio	Accurate determination of phosphorylation occupancy at key p62 sites (e.g., S403/S407)	Cannot obtain absolute stoichiometry of modification sites	[192, 193]
Quantitative thiol reactivity platform	Commercial IPM probe labels reduced cysteine thiols; click chemistry‐isotope labeling‐MS	Mapping reactivity changes of p62 cysteine residues (e.g., C105, C113, C331) under oxidative stress	Quantification and functional site identification of cysteine oxidation modifications	[194]
EXoO	Protease OpeRATOR cleaves N‐terminally to O‐GalNAc‐modified Ser/Thr, ensuring unambiguous site localization	Theoretically applicable to identify O‐GalNAc glycosylation sites on p62 (not yet reported)	Difficulty in precise localization of O‐GalNAc sites	[195]
PBSP	Bioorthogonal probes (e.g., ITalk) covalently label metabolic modifications in live cells; click chemistry enrichment	Screening whether p62 and its interaction network undergo novel metabolic modifications (e.g., itaconation) during immunometabolic reprogramming	Lack of enrichment tools for novel metabolic modifications (e.g., itaconation)	[196]
Single‐molecule nanopore	Electro‐osmotic flow drives full‐length protein through a nanopore; direct reading of PTMs on the polypeptide chain	Holds promise for label‐free, enrichment‐free, single‐molecule detection of native p62 modifications	Cannot directly detect native full‐length protein modifications	[197]
CRISPR/Cas9 gene editing	Site‐directed mutagenesis, knock‐in, knockout combined with proteomics and functional analysis	Deciphering the function of p62 PTMs in physiological and pathological contexts	Cannot establish causality between specific modifications and function in the native environment	[198, 199, 200]

*Abbreviations*: NO‑IP/MS, native organelle immunoprecipitation/mass spectrometry; CLASP, cross‑linking assisted spatial proteomics; EAD‑PRM, electron‑activated dissociation parallel reaction monitoring; EXoO, exoprotease for O‑glycosylation; LC–TIMS–ToF, liquid chromatography trapped ion mobility spectrometry time‑of‑flight; microPhos (µPhos), microplate‑based phosphoproteomics; PBSP, probe‑based bioorthogonal labeling and mass spectrometry; STO‑MS+, stoichiometry mass spectrometry plus; TMT, tandem mass tag.

### 5.3 Toward Therapeutic Translation of the p62 PTMs Network

To dynamically link the intracellular molecular mechanisms of p62 with systemic physiology and pathological states, and ultimately achieve clinical translation, it is necessary to establish a clear research pathway extending from biomarker discovery and validation to the design of precision intervention strategies (Figure 6).

**FIGURE 6:**
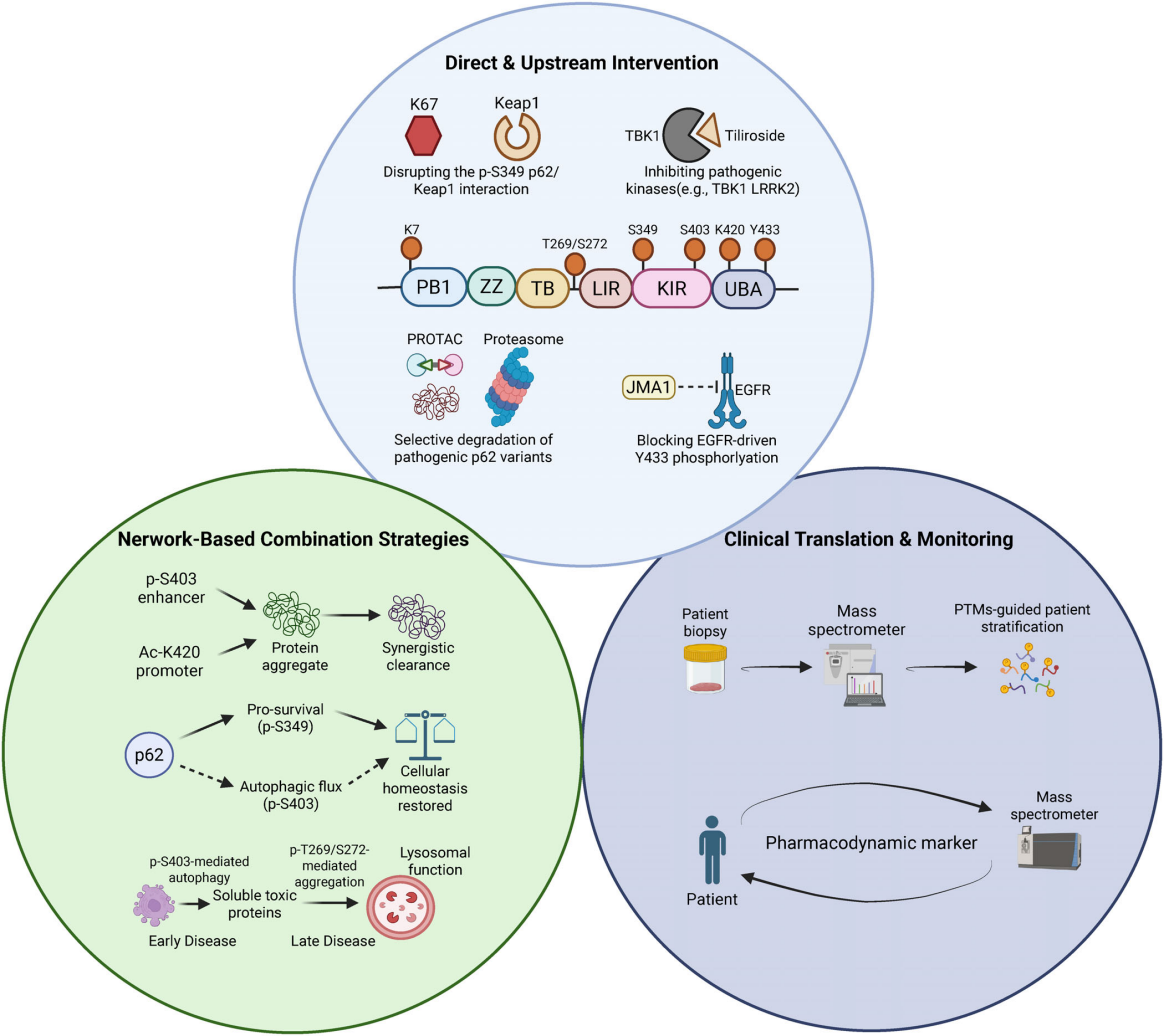
Future therapeutic strategies targeting the p62 PTMs network. Direct and upstream intervention (top): pharmacological modulation of specific PTMs, including: (i) small molecules (e.g., K67) disrupting p‐S349 p62–Keap1 binding; (ii) kinase inhibitors (e.g., tiliroside) targeting TBK1; (iii) stapled peptides (JMA1) blocking EGFR‐driven Y433 phosphorylation; and (iv) PROTACs for selective degradation of pathogenic p62 variants. Network‐based combination strategies (bottom‐left): systems‐level reprogramming to restore proteostasis. Strategies include: (i) synergistic clearance via p‐S403 and K420 acetylation; (ii) homeostatic balancing of p‐S349 prosurvival signaling versus p‐S403‐mediated autophagy; and (iii) stage‐specific intervention, promoting p‐S403‐driven clearance in early disease while enhancing lysosomal function in late‐stage pathology. Clinical translation and monitoring (bottom‐right): a PTMs‐centric precision medicine paradigm. Patient biopsies and mass spectrometry enable PTM‐guided stratification, while real‐time monitoring of circulating markers (e.g., serum p‐S349) facilitates dynamic treatment adjustment. This framework shifts focus from global p62 inhibition to the precise correction of disease‐associated modification patterns.

Building a reliable p62‐specific PTMs biomarker system requires a systematic translational path from clinical clue discovery to clinical utility assessment. First, candidate modifications must be identified and validated based on strong correlative evidence. For instance, phosphorylation of p62 at S349 is consistently and significantly elevated in the brain tissue of AD patients and is specifically enriched within NFTs [117], suggesting its potential as a marker for disease‐associated pathological states. However, p62 phosphorylation modifications, including p‐S349, have not yet been widely applied in the clinical diagnosis or prognosis of AD or other disorders, and further studies are required to confirm their feasibility. Subsequently, unbiased screening and quantitative verification of this modification's prevalence and disease specificity should be conducted using cohort samples with precise clinicopathological annotations, combined with technologies such as ultra‐sensitive phosphoproteomics or specific modification antibodies. To advance toward noninvasive clinical detection, deep endogenous phosphopeptide profiling techniques [201] can be employed to attempt to capture corresponding circulating signature peptides in biofluids like serum or cerebrospinal fluid from matched patients, establishing a crucial bridge connecting histopathology with liquid biopsy. Following this, the definition of this biomarker and its clinical utility should be clarified and evaluated in prospective studies. Validated candidate biomarkers must enter a rigorous stage of clinical utility evaluation, with their application scenarios clearly defined. Examples include sensitivity and specificity for early diagnosis, the ability to determine disease subtype and prognosis, utility as a pharmacodynamic indicator for monitoring treatment response, or as a companion diagnostic tool for screening beneficiary populations. A complete translational example could involve discovering abnormal elevation of p62 S349 phosphorylation in tumor tissue; confirming its association with poor prognosis through validation in an independent tissue cohort and successfully establishing a serum detection method; and finally, using it as a biomarker in clinical trials to guide precision therapy targeting this pathway. This rigorous multistage process ensures the robustness and application value of p62 PTMs biomarkers as they progress from laboratory discovery to clinical practice.

Second, the preclinical studies discussed above have primarily focused on interventions targeting a single PTM site, providing proof‐of‐concept for the targetability of the p62 PTMs network. However, these single‐target strategies are insufficient to address the dynamically changing PTMs network states of p62 during disease progression, as exemplified during proteasome inhibitor resistance in tumors, where p62 can dynamically switch its functional output among T269/S272 phosphorylation, S403 phosphorylation, and S349 phosphorylation to provide tumor cells with multilayered proteotoxic defense [57, 58, 59, 102, 103], and targeting any single modification node alone fails to block all escape routes, highlighting the necessity of combined intervention. Based on this, we further propose three potential future network‐based intervention strategies. The first is synergistic activation or inhibition of pathways, which involves cotargeting multiple nodes in the same functional direction to produce additive or synergistic effects. For example, p62‐mediated selective autophagic clearance of toxic aggregates is a multistep process in which S403 phosphorylation enhances the recognition capacity of the UBA domain for ubiquitinated substrates [43], while K420 acetylation promotes efficient substrate concentration by disrupting UBA dimerization and driving liquid–liquid phase separation [2], and these two modifications act on consecutive but functionally complementary steps—substrate recognition and substrate concentration—so that theoretically, simultaneous enhancement of upstream S403 phosphorylation and downstream K420 acetylation could synergistically improve the overall efficiency of toxic aggregate clearance from both recognition and concentration perspectives, producing a superior effect compared with single‐target intervention, although this hypothesis requires further validation in specific disease models through simultaneous modulation of both modification sites. The second strategy is bidirectional restoration of homeostasis, which involves simultaneously targeting multiple functionally different dysregulated nodes within the network to push the pathological network back toward physiological equilibrium from multiple directions. For instance, in tumor, p62 may persistently activate the Keap1–Nrf2 prosurvival pathway via S349 phosphorylation [11], while simultaneously maintaining excessive autophagic flux via S403 phosphorylation to maintain proteostasis [45, 46]. In this scenario, a combination of a p62–Keap1 interaction inhibitor and a TBK1 kinase inhibitor could simultaneously disrupt aberrant survival signaling and metabolic support, delivering a dual strike against tumor cells with potential synergistic antitumor effects. The third strategy is temporal or sequential intervention, which adjusts therapeutic focus sequentially according to the dynamic evolution of the primary pathological features at different disease stages. During the pathological progression of neurodegenerative diseases, the functional role of p62 is not static, as in the early stage, soluble toxic proteins such as TDP‐43 oligomers constitute the major proteotoxic burden, and cells initiate selective autophagy through TBK1‐mediated p62 S403 phosphorylation to enhance UBA domain recognition of ubiquitinated substrates [28, 43], but as the disease progresses, sustained autophagic pressure and proteostatic imbalance lead to the gradual predominance of insoluble inclusions such as TDP‐43 pathological aggregates in ALS or NFTs in AD [111, 119], and at this stage, excessive or persistent S403 phosphorylation paradoxically drives the formation of hyperstable, autophagy‐resistant aggregates, exacerbating neurotoxicity [108], whereas T269/S272 phosphorylation drives p62 to encapsulate toxic proteins into relatively inert aggregates for emergency sequestration [57, 58]. Therefore, based on this dynamic evolution from soluble aggregates to insoluble inclusions, a sequential intervention strategy emerges: the early intervention focus should be to enhance p62‐mediated selective autophagy, for example, by promoting S403 phosphorylation, to clear soluble toxic proteins, while the late‐stage strategy should shift toward utilizing p62's aggregation function for toxicity sequestration, for example, by modulating T269/S272 phosphorylation, combined with enhancing lysosomal clearance capacity to dispose of already formed insoluble inclusions. This dynamic therapeutic concept reflects a deep understanding of the spatiotemporal regulation of the p62 PTMs network.

Building upon the foundation laid by existing intervention strategies, the core future task is to comprehensively translate the mechanistic understanding of pathogenic p62 PTMs into a multilayered, precisely controllable targeted therapy system. The evolutionary direction is to deeply expand from intervening in the modification events themselves toward programming the functional state of the network. On one hand, based on structural biology, small molecules or cyclic peptide inhibitors can be designed to precisely recognize abnormal protein–protein interaction interfaces created or exposed by modifications such as phosphorylation and ubiquitination, achieving spatial blockade of specific pathological interactions. On the other hand, for p62 variants that have gained function or formed stubborn aggregates due to abnormal modifications, novel degradation technologies like PROTAC [202] can be leveraged to develop bifunctional molecules that specifically recognize the modification‐dependent conformation. This would enable the selective clearance of these pathogenic variants, helping to overcome challenges such as protein aggregation and sustained activation that are difficult for traditional drugs to address. Additionally, developing allosteric modulators to reset p62's functional balance has a successful precedent. For example, allosteric modulators for the dysfunctional Parkin protein in PD can act as molecular glues to stabilize its active conformation, thereby restoring its function [203].

In summary, translating the dynamic regulatory atlas of the p62 PTMs network into a clinically applicable precision medicine system represents the core objective and ultimate challenge for future research [204]. Achieving this goal relies on establishing a closed‐loop paradigm encompassing the entire chain of detection, targeting, and monitoring. First, it is necessary to identify and validate disease‐specific p62 PTMs molecular subtypes within multidimensional clinical cohorts, leveraging high‐throughput proteomics, structural biology, and computational modeling. This will establish their role as biomarkers for diagnostic stratification and prognostic assessment. Subsequently, based on the network imbalance features revealed by different molecular subtypes—such as a prosurvival state dominated by S349 phosphorylation or an autophagy‐dysregulated state stemming from K420 multimodification imbalance—advanced strategies like synergistic activation, bidirectional restoration, or temporal intervention can be precisely matched and applied to reprogram the network state. Throughout this process, these PTMs features themselves can serve as real‐time pharmacodynamic indicators for dynamically assessing therapeutic response and guiding regimen adjustments.

However, deciphering this complex network and achieving translation is inherently a highly interdisciplinary systems engineering project. It requires structural biology to elucidate the precise conformational regulatory mechanisms of PTMs, chemical biology to develop specific modification‐regulating tools and probes, and computational biology to build predictive dynamic network models to guide combination therapies, all ultimately validated through cutting‐edge clinical research for their translational value and utility. Only through such deep interdisciplinary integration can personalized treatment based on an individual patient's p62 PTMs profile be transformed from blueprint to reality. This approach aims to reset pathological homeostasis at the systems level, offering a novel therapeutic paradigm for conquering major complex diseases such as cancer and neurodegenerative disorders.

### 5.4 Concluding Perspectives on p62 in Physiology and Disease

Based on the systematic exposition presented throughout this review, the function of p62 extends far beyond that of a static autophagy adaptor protein. This review reveals its essence as a molecular processor meticulously programmed by a complex and dynamic PTMs network. Under physiological homeostasis, this network integrates multidimensional signals—such as nutrients, oxidative stress, metabolism, and immunity—by employing reversible, coordinated, and spatiotemporally specific modifications, including phosphorylation, ubiquitination, and acetylation. These signals are encoded into distinct p62 PTMs patterns, which in turn precisely regulate core cellular processes like autophagic flux, antioxidant defense, cell growth, genome maintenance, and inflammatory responses. This exemplifies the high adaptability and integrative capacity of cells in responding to environmental challenges.

However, in pathological states such as cancer, neurodegenerative diseases, metabolic syndromes, and infections, sustained aberrant signaling or genetic mutations can lead to a pathological reprogramming of this precise network, disrupting its homeostatic coding. This manifests concretely as: reversible regulatory switches becoming persistently activated driver switches, exemplified by oncogenic signal‐locked S349 phosphorylation in tumors; precise spatiotemporal control devolving into dysfunctional or contradictory fault points, such as the inhibition of aggregate clearance by S403 phosphorylation in neurodegenerative diseases; and flexible signal integration capacity deteriorating into rigid, imbalanced signal output. This dysregulation of the PTMs network represents the common molecular basis for p62's transformation from a homeostasis maintainer into a disease driver, a process that permeates the entire course of disease initiation, progression, and therapeutic resistance.

Consequently, the research paradigm for p62 must undergo a fundamental shift. Its core value lies not in being a static protein target, but in its dynamic PTMs functional state. This insight unveils a novel therapeutic logic: the goal should be targeting specific PTMs codes rather than the p62 protein itself. Looking ahead, a path toward revolutionary diagnostics and therapies for major refractory diseases like cancer and neurodegenerative disorders will be paved by interdisciplinary efforts to systematically decipher the dynamic atlas of the PTMs network, develop tools capable of real‐time monitoring and precise editing of specific pathological PTMs codes, and design intelligent intervention strategies based on this knowledge. Ultimately, the vision for this field is the realization of precision medicine based on an individual's p62 PTMs profile.

## Author Contributions

Lifeng Feng, Hongchuan Jin, and Liyuan Zhu designed the study. Weikai Wang, Xin Jiang, Runing Qiu, Chandra Sugiarto Wijaya, Miaoqin Chen, and Jin Wang wrote the manuscript; All authors read and approved the final manuscript.

## Funding

This work was supported by the National Natural Science Foundation of China (No. 82273092) and Natural Science Foundation of Zhejiang Province (No. LZ24H160006, LY22H160001, LQ22H160060, and LY24H160011).

## Ethics Statement

The authors have nothing to report.

## Conflicts of Interest

The authors declare no conflicts of interest.

## Supporting information




**Supporting File 1**: mco270949‐sup‐0001‐FigureS1.docx

## Data Availability

The authors have nothing to report.
