# Supplementary material for: Posttranslational Modifications of p62/SQSTM1 in Health and Disease
Source: MedComm (2020). 2026 Sep 30;7(10):e70949. doi: 10.1002/mco2.70949 (PMC13625819; doi:10.1002/mco2.70949)
Supplement: Supplementary file 1 [file MCO2-7-e70949-s001.docx]

Post-translational modifications of p62/SQSTM1 in health and disease

Weikai Wang^1#^, Xin Jiang^1#^, Runing Qiu^1^, Chandra Sugiarto Wijaya^1^, Miaoqin Chen^1^, Jin Wang^2^, Liyuan Zhu^1*^, Hongchuan Jin^1*^, Lifeng Feng^1*^

1 Department of Medical Oncology, Zhejiang Key Laboratory of Multi-omics Precision Diagnosis and Treatment of Liver Diseases, Cancer Center of Zhejiang University, Sir Run Run Shaw Hospital, School of Medicine, Zhejiang University, Hangzhou, Zhejiang, China

2 Department of Pathology, Sir Run Run Shaw Hospital, School of Medicine, Zhejiang University, Hangzhou, Zhejiang, China

Correspondence: Lifeng Feng, Hongchuan Jin and Liyuan Zhu, Department of Medical Oncology, Zhejiang Key Laboratory of Multi-omics Precision Diagnosis and Treatment of Liver Diseases, Cancer Center of Zhejiang University, Sir Run Run Shaw Hospital, School of Medicine, Zhejiang University, Hangzhou, Zhejiang, China. Email: lffeng@zju.edu.cn, jinhc@zju.edu.cn, zly_smile@126.com

These authors contributed equally: Weikai Wang, Xin Jiang

**Supplementary Figure and Legend**

**Figure S1. Domain and PTMs functional map of p62/SQSTM1.** Schematic representation of p62/SQSTM1 structural domains and their regulatory PTMs. Upstream modifying/de-modifying enzymes are annotated adjacent to specific residues, illustrating how PTMs orchestrate key functional outcomes, including selective autophagy, the Keap1-Nrf2 antioxidant response, and disease-related signaling. Panels (A–E) are arranged according to the domain regions, depicting PTM sites and regulatory enzymes along the p62 protein sequence.
